# Genetic variation in *GC* and *CYP2R1* affects 25-hydroxyvitamin D concentration and skeletal parameters: A genome-wide association study in 24-month-old Finnish children

**DOI:** 10.1371/journal.pgen.1008530

**Published:** 2019-12-16

**Authors:** Anders Kämpe, Maria Enlund-Cerullo, Saara Valkama, Elisa Holmlund-Suila, Jenni Rosendahl, Helena Hauta-alus, Minna Pekkinen, Sture Andersson, Outi Mäkitie

**Affiliations:** 1 Department of Molecular Medicine and Surgery, Karolinska Institutet, Stockholm, Sweden; 2 Department of Clinical Genetics, Karolinska University Hospital, Stockholm, Sweden; 3 Center for Molecular Medicine, Karolinska Institutet, Stockholm, Sweden; 4 Children’s Hospital, Pediatric Research Center, University of Helsinki and Helsinki University Hospital, Helsinki, Finland; 5 Folkhälsan Research Center, Helsinki, Finland; 6 Research Program for Clinical and Molecular Metabolism, Faculty of Medicine, University of Helsinki, Finland; McGill University, CANADA

## Abstract

Vitamin D is important for normal skeletal homeostasis, especially in growing children. There are no previous genome-wide association (GWA) studies exploring genetic factors that influence vitamin D metabolism in early childhood. We performed a GWA study on serum 25-hydroxyvitamin D (25(OH)D) and response to supplementation in 761 healthy term-born Finnish 24-month-old children, who participated in a randomized clinical trial comparing effects of 10 μg and 30 μg of daily vitamin D supplementation from age 2 weeks to 24 months. Using the Illumina Infinium Global Screening Array, which has been optimized for imputation, a total of 686085 markers were genotyped across the genome. Serum 25(OH)D was measured at the end of the intervention at 24 months of age. Skeletal parameters reflecting bone strength were determined at the distal tibia at 24 months using peripheral quantitative computed tomography (pQCT) (data available for 648 children). For 25(OH)D, two strong GWA signals were identified, localizing to *GC* (Vitamin D binding protein) and *CYP2R1* (Vitamin D 25-hydroxylase) genes. The GWA locus comprising the *GC* gene also associated with response to supplementation. Further evidence for the importance of these two genes was obtained by comparing association signals to gene expression data from the Genotype-Tissue Expression project and performing colocalization analyses. Through the identification of haplotypes associated with low or high 25(OH)D concentrations we used a Mendelian randomization approach to show that haplotypes associating with low 25(OH)D were also associated with low pQCT parameters in the 24-month-old children. In this first GWA study on 25(OH)D in this age group we show that already at the age of 24 months genetic variation influences 25(OH)D concentrations and determines response to supplementation, with genome-wide significant associations with *GC* and *CYP2R1*. Also, the dual association between haplotypes, 25(OH)D and pQCT parameters gives support for vertical pleiotropy mediated by 25(OH)D.

## Author summary

The effect of vitamin D continues to be highly debated in various health outcomes, including bone health. In this first study of children this young we searched for genes that modify vitamin D metabolism in early childhood using a genome-wide analysis of almost 700,000 genetic variants in a cohort of 761 healthy children participating in a vitamin D intervention study. We show that genetic variation in the genes coding for Vitamin D binding protein (*GC*) and Vitamin D 25-hydroxylase (*CYP2R1*) are important determinants for serum 25-hydroxyvitamin D concentration in 2-year-old children. Genetic variants within the *GC* gene also affect how the child responds to vitamin D supplementation. Moreover, our findings suggest that in 2-year-old children vitamin D concentration, even when within the normal range, influences bone strength as children with genetic constellations associating with lower vitamin D concentration and poorer response to vitamin D supplementation also have weaker bones.

## Introduction

Vitamin D is a fat-soluble prohormone essential for calcium and phosphate homeostasis, but also believed to be important for many other cellular processes in the human body. Vitamin D deficiency has been associated with various diseases and outcomes, including skeletal disorders, infections, autoimmunity and all-cause mortality [1, 2]. However, in large systematic reviews and randomized trials, the effects of vitamin D on human health have been varying and associations to health outcomes hard to replicate [3–5]. The best understood consequences of vitamin D deficiency are rickets in children and osteomalacia in adults, two disorders characterized by poor mineralization of the bone matrix [6, 7]. Especially for the growing skeleton during childhood, sufficient absorption of calcium and phosphate, mainly mediated by active vitamin D, is important. Since vitamin D synthesis in the skin from 7-dehydrocholesterol requires sun light exposure, children living in the northern latitudes have a particularly high risk for vitamin D deficiency [8–10]. Vitamin D food fortification and supplementation have been implemented to improve vitamin D status in these countries, including Finland [10, 11].

We hypothesized that individual genetic properties may be of great importance in the maintenance of vitamin D sufficiency and that these genetic properties and their effects may be more easily identified in Finnish children whose sunlight exposure is low. In conjunction with a randomized vitamin D intervention study comparing effects of 10 μg and 30 μg of daily vitamin D supplementation from age 2 weeks to 24 months [12], we performed a genome wide association (GWA) study. The GWA study focused on 25-hydroxy vitamin D (25(OH)D) concentrations at the 24-month time-point, to search for genetic variations that are important determinants for 25(OH)D concentration in 24-month-old healthy Finnish children. The 25(OH)D concentration at 24 months was chosen as the outcome variable, although 25(OH)D had also been assessed at birth (umbilical cord) and at 12 months. At 24 months the mother’s 25(OH)D is less likely to have an influence [13] and the feeding patterns are more constant after decreased influence of breast feeding. We adjusted all association models for intervention group because of its strong effect on 25(OH)D at the 24-month time point, but having two intervention groups also enabled us to assess genetic variation in relation to supplementation response. We further explored how genetic variation associating with either higher or lower 25(OH)D concentration affected skeletal parameters measured with peripheral quantitative computed tomography (pQCT). Although several previous GWA studies have explored genetic factors associating with 25(OH)D in adults and older children, this is the first study looking at genome wide genetic variation in relation to 25(OH)D concentration and skeletal outcomes in this age group of 2-year-old children.

## Results and discussion

All participants in this study were originally included in the Vitamin D intervention (VIDI) study, a randomized clinical trial investigating whether 30 μg compared with 10 μg of daily vitamin D_3_ supplementation, given from age 2 weeks to 24 months, would be beneficial for Finnish infants [12] (also see Methods). The 25(OH)D concentration at 24 months was chosen to best represent the children’s inherent 25(OH)D concentrations. For the GWA test the 25(OH)D concentration was treated as a continuous variable and the applied linear model was adjusted for randomization group, sex, season and the first 4 principal components. In order to maximize genetic homogeneity in the cohort, we performed an ancestry analysis in which we excluded 22 individuals because they did not cluster close enough to the 99 Finnish reference individuals encompassed within the 1000 genomes project [see Methods].

### Two genome-wide significant loci near the genes *GC* and *CYP2R1*

**Fig 1:**
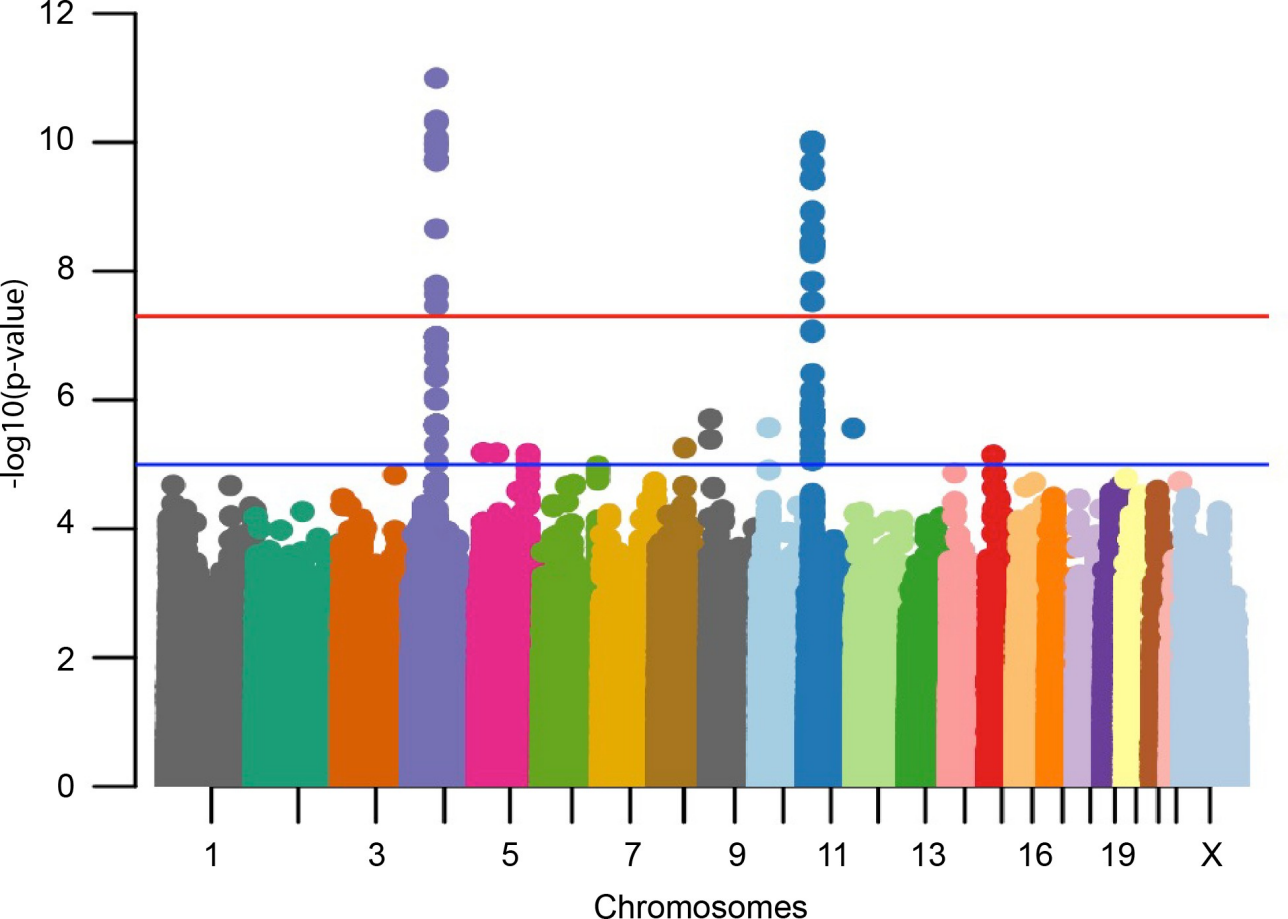
Association results for 25(OH)D concentration in 24-month old children. The results show two loci, one on chromosome 4 and one on chromosome 11, that are strongly associated to serum 25(OH)D concentration in 24-months old children. The genomic inflation factor (λ) for the association test is very close to 1 (ℷ = 0.997), suggesting a solid dataset where the association statistics are not inflated due to population stratification or poor-quality data.

**Fig 2:**
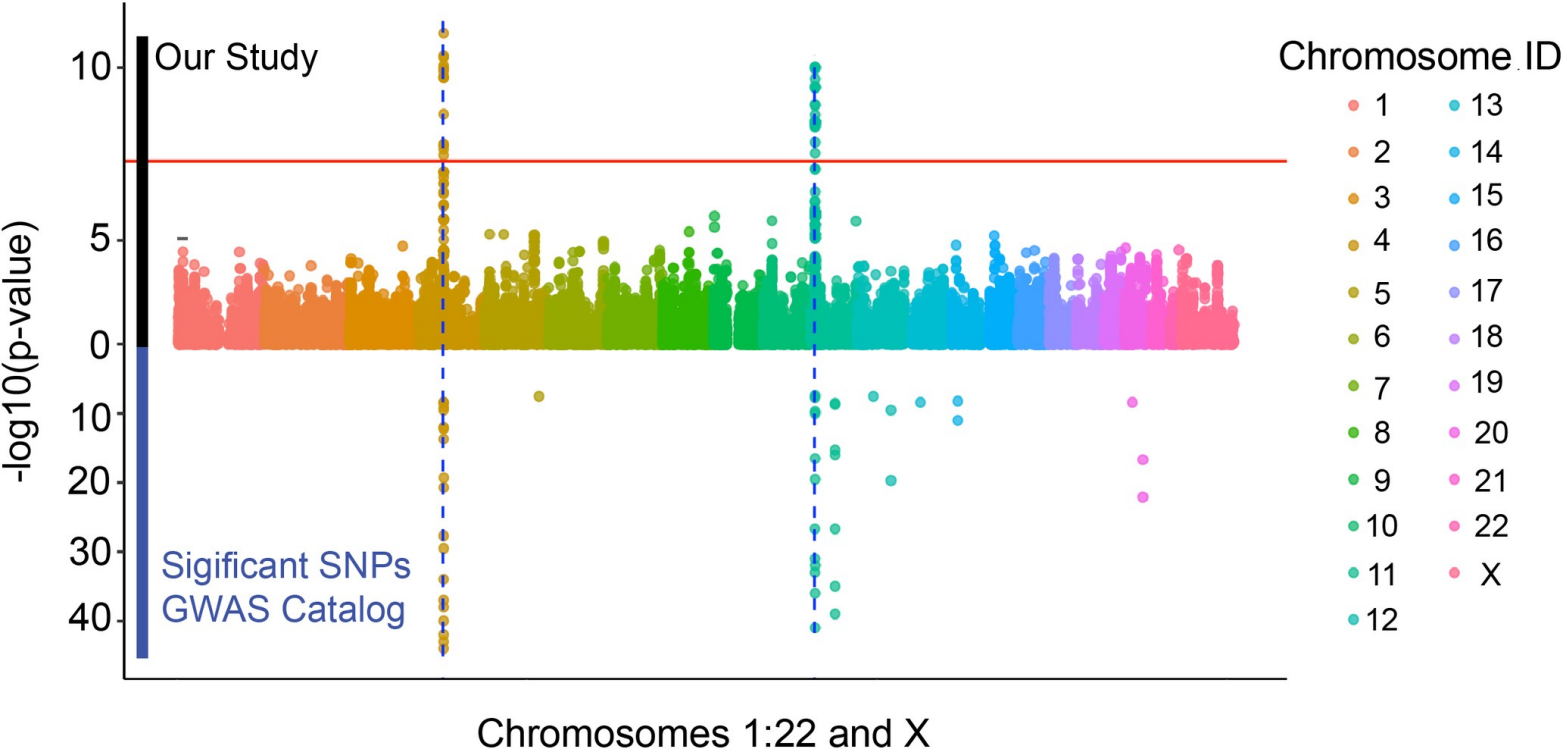
Significant associations in our study vs the GWAS catalog. Association results from our study on 25(OH)D are displayed above the line y = 0, while statistics from the GWAS catalog are displayed below it. All SNPs reported to the GWAS catalog that have been significantly associated (genome-wide) to the trait 25(OH)D have been included. The two loci associated to 25(OH)D in 24-month old children are located within the two strongest previously reported loci. The y-axis has been distorted due to graphical reasons.

Altogether 928 children participating in the original intervention study were genotyped using Illumina’s Infinium Global Screening Array. Of them, 761 had both genotype data that passed all quality control filters [see Methods] and a 25(OH)D measurement for the 24-month timepoint. Anthropometric, biochemical and skeletal measurements are shown in Table 1. The GWA results showed two strong signals that surpassed the genome-wide significance threshold (p≤5x10^-8^), one on chromosome 4 and one on chromosome 11 (Fig 1, QQ-plot in S1 Fig). The genomic inflation factor for the association test was 0.997, suggesting a genetically homogenous dataset with few spurious associations. The lead SNP on the chromosome 4 locus, rs1155563 (p = 1.011 x10^-11^), was a non-imputed SNP in the first intron of the gene *GC* (Vitamin D binding protein; ENST00000273951.8). For the signal seen on chromosome 11, the lead SNP rs10832310 (p = 4.241 x10^-11^) was located in intron 12 of the *PDE3B* gene (Phosphodiesterase 3B; ENST00000282096.4), but also in close proximity to *CYP2R1* (Vitamin D 25-hydroxylase; ENST00000334636.5). Both these two loci have in previous GWA studies been shown to associate with serum 25(OH)D but no GWA data exist for children as young as our cohort. We identified in the GWAS catalog 9 separate studies using 25(OH)D concentration as the studied trait (accessed September 2019) [14–23]. These 9 studies reported in total 47 independent SNPs (41 unique SNPs) that were associated with 25(OH)D levels on a genome wide significant level (Fig 2). The two signals identified in our study are located within the two strongest previously known loci for 25(OH)D concentrations, most often mapped to the genes *GC* and *CYP2R1* [14, 16, 17, 19–21]. However, in a pediatric setting, the GWAS catalog only reports one previous GWA-study on 25(OH)D, involving children aged ≥6 years, that has been able to find genome-wide significant loci [20].

**Table 1:** Anthropometric, biochemical and skeletal parameters in the cohort.

	Boys (n = 383, 50.3%)		Girls (n = 378, 49.7%)		Boys vs Girls
Parameter	Mean value (SD)	Missing (n)	Mean value (SD)	Missing (n)	p-value
Birth weight (kg)	3.6 (0.38)	-	3.5 (0.39)	-	<0.001
Birth length (cm)	50.6 (1.7)	-	50.0 (1.7)	-	<0.001
Height at 24 months (cm)	88.6 (3.1)	1	86.9 (2.8)	-	<0.001
Weight at 24 months (cm)	12.9 (1.4)	3	12.1 (1.3)	-	<0.001
Length adjusted weight at 24 months (SD)	-0.02 (0.99)	3	-0.12 (0.96)	-	0.17
25(OH)D—at birth (nmol/L)	84.9 (27.5)	2	80.0 (24.0)	5	0.009
25(OH)D—24 months (nmol/L)	100.6 (27.6)	-	103.0 (27.9)	-	0.23
Group_10_ vs Group_30_ (number)	195 vs 188	-	190 vs 188	-	0.75 / 0.96
Group_10_ vs Group_30_−25(OH)D at 24 months (nmol/L) (SD)	88.0 (19.5) vs 118.2 (27.0)	-	85.1 (20.0) vs 116.7 (25.0)	-	-
	Boys (n = 309, 47.7%)		Girls (n = 339, 52.3%)		Boys vs Girls
pQCT measurements at 24 months	Mean value (SD)		Mean value (SD)		p-value
Total bone mineral density (mg/cm^3^)	380.0 (75.4)		374.3 (76.2)		0.34
Total bone mineral content (mg/mm)	56.1 (8.1)		52.8 (7.6)		<0.001
Total bone area (mm^2^)	151.4 (27.7)		145.1 (27.4)		0.004
Cortical mineral density (mg/cm^3^)	727.3 (61.8)		726.2 (62.6)		0.83
Cortical mineral content (mg/mm)	44.6 [9.2)		41.6 (9.1)		<0.001
Cortical area (mm^2^)	61.0 (9.1)		56.8 (9.2)		<0.001

**Fig 3:**
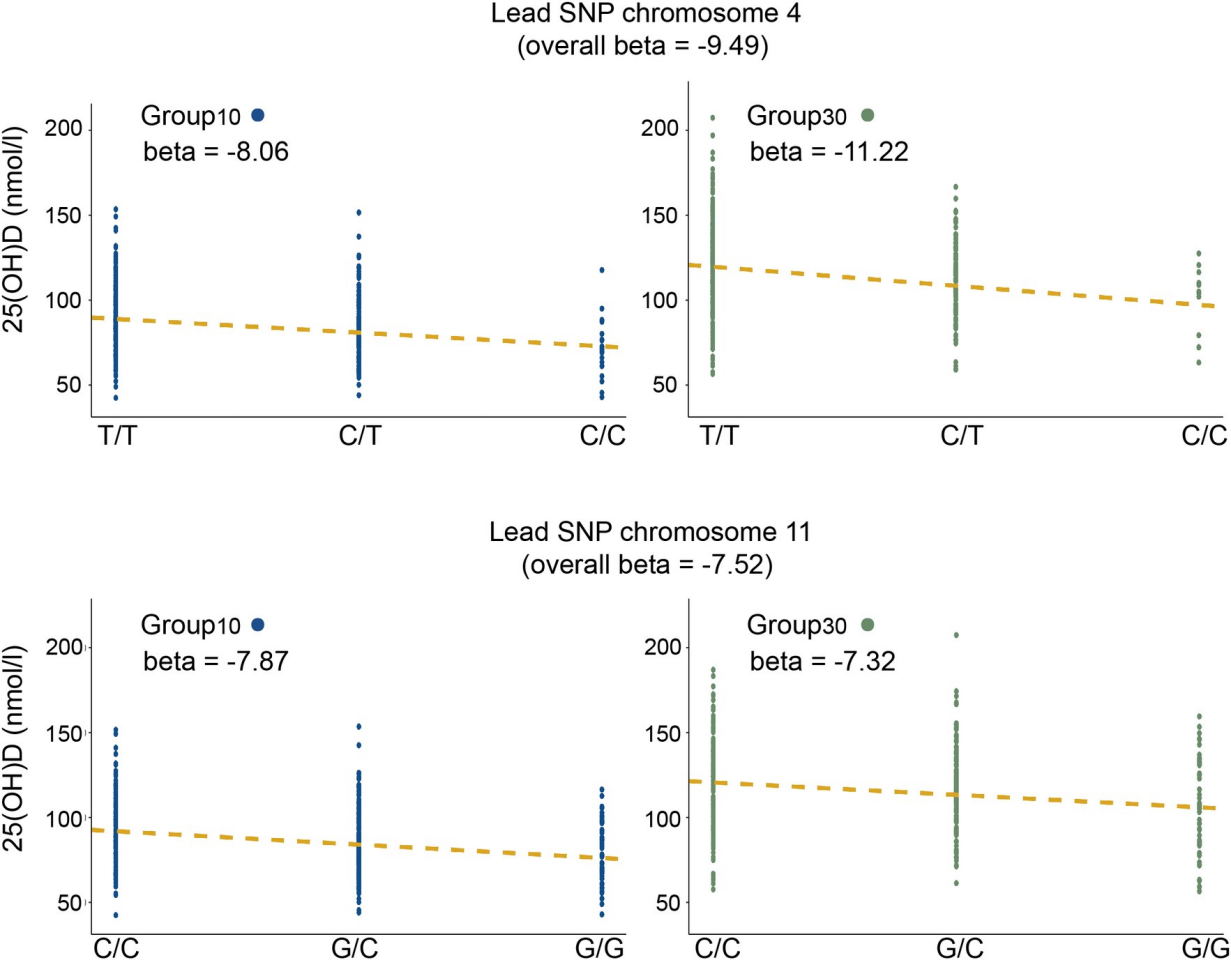
Association statistics for Group_10_ vs Group_30_. The analysis focuses on the lead SNPs at each significantly associated locus while separating the randomization groups. The results show that the lead SNP on chromosome 4 (rs1155563) seem to have a larger effect in the group receiving 30 μg of daily vitamin D (Group_30_) than in the group receiving 10 μg (Group_10_). The lead SNP on chromosome 11 (rs10832310) instead displayed a similar effect size regardless of randomization group, suggesting that the locus on chromosome 4 can modulate supplementation response, while the locus on chromosome 11 does not. Y-axis is 25(OH)D at 24 months.

At 24 months we observed no difference between boys and girls in their overall 25(OH)D level. Further, sex did not have a significant effect on the association strength between either of the two lead SNPs and 25(OH)D (p = 0.15 for rs1155563 and p = 0.1111 for rs10832310)). However, the intervention had a strong effect on 25(OH)D concentrations at 24 months in the 761 analyzed individuals (beta = 30.88, p<2x10^-16^). Because of this strong effect we wanted to further investigate the randomization group’s contribution. We performed the same association analysis after separating the two groups receiving 10 μg or 30 μg vitamin D daily (denoted Group_10_ and Group_30_). The effect allele (C) at the lead SNP on chromosome 4 (rs1155563) appeared to have a larger negative effect on 25(OH)D in Group_30_ compared to Group_10_, while the lead SNP on chromosome 11 (rs10832310) showed a similar effect size regardless of the randomization group (Fig 3). However, no significant interaction effects between the lead SNPs and the randomization group were observed (rs1155563: beta = -3:12, p = 0.259; rs10832310: beta = 0.1987, p = 0.930). Exclusion of the randomization group from the model weakened the association with 25(OH)D for the chromosome 11 lead SNP (p = 1.37^−09^ vs p = 4.24^−11^), which was expected. However, for the chromosome 4 lead SNP (rs1155563) we now saw a larger difference in mean 25(OH)D between the genotypes and the association became stronger (p = 2.16^−13^ vs p = 1.01^−11^). This was unexpected, because the randomization group should be considered as a competing exposure variable, not a confounder, and exclusion of such a variable should weaken the model. The observed strengthening of the association suggested that a part of the signal on chromosome 4 may be explained by the intervention effect, meaning that children carrying the allele associated with low 25(OH)D respond less efficiently to vitamin D supplementation.

This observation motivated us to further evaluate how the genotypes affected the response to vitamin D intervention during the controlled trial. We constructed a random intercept linear mixed model using the lme4 R package. The 25(OH)D concentrations at two time points, (1) at birth (umbilical cord) and (2) at 24 months, were used as outcome. The model was adjusted for sex and the mother’s 25(OH)D concentration during pregnancy, which impacts cord blood 25(OH)D, (data available for 648 mothers). Since no subjects had received any vitamin D supplementation at the first time point (birth), the interaction effect between genotype and time was assessed separately in the two randomization groups. A significant interaction effect between genotype and time was only seen in the intervention group (Group_30_) for the lead SNP on chromosome 4 (rs1155563, p = 0.02798, interaction effect: genotype:time: -7.40596). These findings imply that the effect allele (C) of rs1155563, associated with low 25(OH)D, also reduces response to high dose vitamin D supplementation (S1 Appendix).

### *GC* and *CYP2R1* are likely to cause the observed association signals

**Fig 4:**
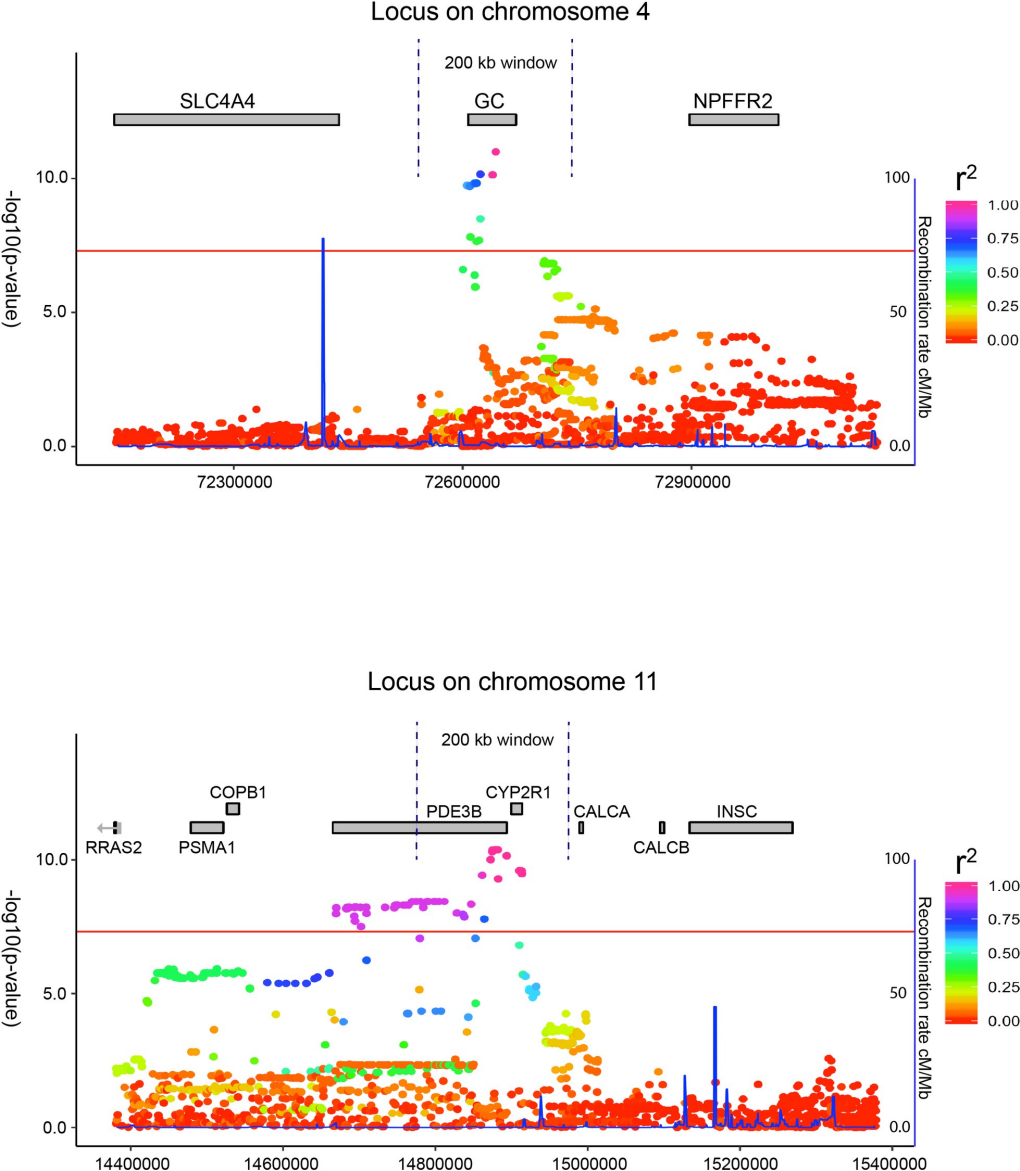
The two loci and their nearby genomic regions. A close up view of the genomic regions immediately surrounding the lead SNPs in each locus. The gene *GC* is the only gene within the 200 kb target window on chromosome 4, making it the likely candidate for the signal. For the locus on chromosome 11, both *PDE3B* and *CYP2R1* are located within the window for likely candidates as the underlying cause of the signal.

All SNPs in a 5 Mb window around the lead SNP in each locus were re-imputed from non-phased genotypes to increase imputation precision, as recommended by IMPUTE2. Conditioning on the respective lead SNP in both loci completely suppressed the association signals, implying presence of only one independent signal per loci. Fig 4 shows the two associated loci with the top SNPs in relation to the nearby genes. Using a 100 kb window on both sides of the lead SNPs as the presumed resolution, the most likely gene to give the signal on chromosome 4 is the *GC* gene, encoding vitamin D binding protein. Intestinal absorption of vitamin D is inadequately characterized, but the process is no longer thought to be only passive [24]. Vitamin D binding protein is highly expressed in the stomach, duodenum and gallbladder[25], suggesting a role in dietary vitamin D absorption. The protein also has a key role in maintaining 25(OH)D concentrations by mediating its reabsorption in the kidneys [26]. Henderson et al. described recently an adult female with severe vitamin D deficiency due to a homozygous *GC* gene deletion. The patient was also resistant to treatment and despite very large doses of vitamin D supplementation her 25(OH)D was unmeasurable [27]. Vitamin D binding protein may thus play an important role when handling large quantities of oral vitamin D intake, and SNPs affecting its regulation or function could influence the efficiency of this process.

Regarding the signal on chromosome 11, both *PDE3B* and *CYP2R1* lie within this target window. Since *CYP2R1* encodes vitamin D 25-hydroxylase that converts cholecalciferol to 25(OH)D, it can be considered the most likely candidate for the signal. Genetic variation in *CYP2R1* is also known to affect 25(OH)D concentrations, and a rare functional variant (p.Leu99Pro) has been shown to significantly lower 25(OH)D concentrations [28]. The applied 100 kb window is based on studies by Wu et al. who recently quantified the mapping precision of associated SNPs in genome-wide association studies [29]. They showed that the distance between the causal variant and the top associated GWAS SNPs most often is shorter than 25.1 Kb and almost always (≈95%) shorter than 100 Kb if the top associated SNPs are common (MAF≥0.01). Their conclusion derived from extensive simulations on whole-genome sequencing data from 3642 unrelated individuals from the UK10K project. We therefore consider the rationale for using a 100 Kb window to search for underlying causes to be strong, but we also acknowledge that the distance relationship between a causal variant and the causal effector gene can vary widely [30].

### Comparing association signals with gene expression data

In an effort to more confidently map the association signals to specific genes we compared the top 10 associated SNPs, and all non-imputed SNPs that passed the genome-wide significance level for each locus, with the full GTEx dataset (V7) with eQTL data for more than 10 000 samples in 53 different tissues [31]. For the loci on chromosome 4, two SNPs (the lead SNP and the third highest ranked SNP) were significantly associated with an expression change in the gene *GC* for the tissue ‘Stomach’. No other significant associations were seen for any other gene or tissue. For the chromosome 11 locus, 10 of the top-ranking SNPs were significantly associated with an expression change altogether in 5 genes in 9 different tissues. These 10 top-ranking SNPs had a common denominator, they were all significantly associated with the expression level in 3 genes: *CYP2R1* (in Thyroid), *PDE3B* (in Pancreas) and *RRAS2* (Tibial Nerve). This common denominator became our focus for further analysis. We extracted all variants included in the GTEx dataset within the 200 kb target window on chromosome 4 for the tissue ‘Stomach’, and on chromosome 11, for the tissues ‘Thyroid’, ‘Pancreas’ and ‘Tibial Nerve’. Because the analysis was limited to the target windows we readjusted the significance threshold, after intersecting the two datasets, to a false discovery rate (FDR) ≤0.05 [See Methods]. Finally, we made a comparison analysis to look for intersecting significant variants in our association test and the GTEx dataset.

**Fig 5:**
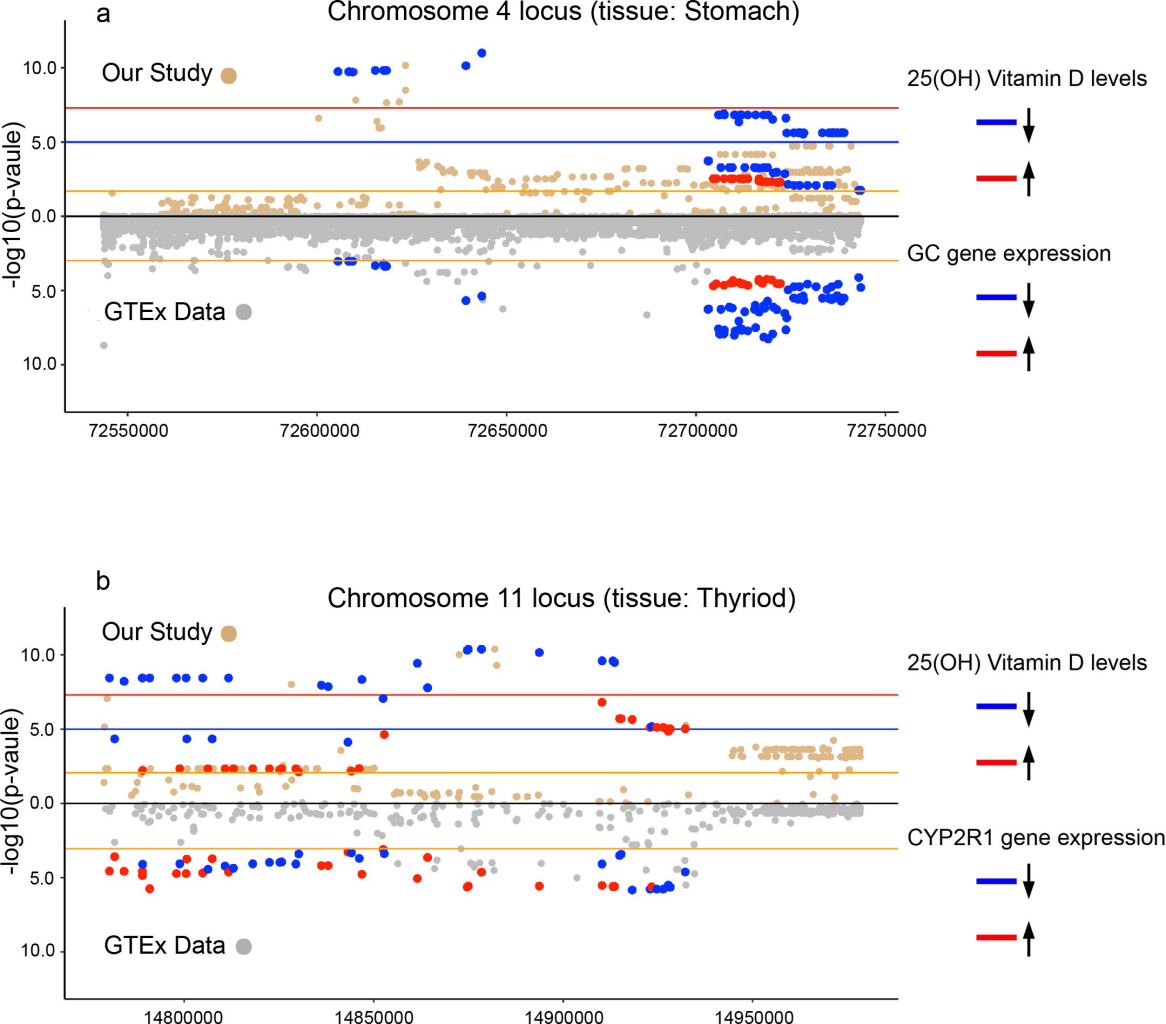
Association statistics for 25(OH)D in 24-month old children vs GTEx expression data. **a)** Association statistics for 25(OH)D in 24-month old children within the 200kb target window on chromosome 4 compared to the GTEx eQTL dataset for the tissue ‘Stomach’. All SNPs that both show a significant association statistic and are present in both datasets are colored either blue (-) or red (+), depending on the direction of the SNP’s beta coefficient. As shown in the figure, all significant SNPs present in both datasets have a concordant direction of association. **b)** Association statistic comparison for SNPs within the target window on chromosome 11, both in our study and in the GTEx dataset for the tissue ‘Thyroid’. Also for the chromosome 11 locus complete concordance in the SNP’s association direction is seen. (red line: genome-wide significance; blue line: genome-wide suggestive significance; orange line: FDR 0.05).

For the 200 kb target window on chromosome 4, our dataset included 237 variants significantly associated with 25(OH)D concentrations (FDR≤0.05). For the same window there were 107 variants in the GTEx dataset (tissue: Stomach) significantly associated with any gene expression changes; 91 of these variants, all associated to a gene expression change in the *GC* gene, intersected in the two datasets, meaning that they were simultaneously associated with *GC* gene expression levels and 25(OH)D. Of the 91 variants, 73 variants (80%) were significantly associated with both a lower expression of *GC* in the GTEx dataset and low 25(OH)D in our study. The remaining 18 variants (20%) were significantly associated with both higher expression of *GC* in GTEx dataset and high 25(OH)D in our study (Fig 5A). In an attempt to quantify the signal similarities between our dataset and the GTEx dataset we used a bayes factor colocalization analysis from the R package “coloc” [32, 33]. This method approximates the posterior probability that a shared variant is the underlying cause of both signals. The analysis was performed on the 91 intersecting variants significantly associated to 25(OH)D and *GC* gene expression, and the results show a posterior probability of 32.8% that the same single variant is causing the association signals in both datasets (S1 Appendix). A caveat is that the method assumes one single causal variant for each trait and the precision of the method is also dependent on the number of significant SNPs used as input. However, together with what is known, these results do suggest that the signal we observed on chromosome 4 can be confidentially mapped to the *GC* gene. The tissue ‘Stomach’ was regarded relevant because of *GC*’s high expression in the stomach and because Vitamin D binding protein mediates the transport of 25(OH)D from the gastrointestinal tract to the liver and other tissues [25, 34, 35].

For the target window in the chromosome 11 locus, the results were not as unequivocal. In the tissue ‘Thyroid’, 52 intersecting SNPs were present. Twenty-seven variants (52%) were simultaneously associated with high *CYP2R1* expression and with low 25(OH)D, whereas the other 25 variants (48%) associated with low *CYP2R1* expression and high 25(OH)D (Fig 5B). For *CYP2R1* these results were opposite to what was expected since we anticipated higher levels of *CYP2R1* to associate with higher 25(OH)D concentration. The biological relevance of 25(OH)D in the Thyroid can also be questioned [36]. However, the complete concordance in the direction of association does further implicate *CYP2R1* as the gene responsible for the association signal in chromosome 11, also because of its definite role in vitamin D metabolism. Regarding the genes *PDE3B* (tissue: Pancreas) and *RRAS2* (tissue: Tibial Nerve), residing in close proximity to the same locus but lacking a clear role in vitamin D metabolism, we again observed a complete concordance in the direction of SNPs’ association with 25(OH)D levels in our study and gene expression in the GTEx dataset (S2 Fig). SNPs associated with 25(OH)D levels within the chromosome 11 locus thus seem to affect the expression of several genes in different tissues, but in a concordant manner relative to 25(OH)D levels. Performing the same colocalization analysis as above for the chromosome 11 locus, the results show strong support for colocalization of the signals in our study and the GTEx dataset. The posterior probability for a shared causal variant was 97.3% for the intersecting SNPs significantly associated to 25(OH)D and *CYP2R1* expression. For the genes *PDE3B* and *RRAS* the results also show a high probability for a shared cause of the signals. The significance of these observations remains unclear, but suggests a more complex co-regulation at the chromosome 11 locus. Although we cannot separate *CYP2R1* from *PDE3B* and *RRAS2* using the results from the eQTL comparison analysis, our data together with previously published studies collectively suggest that *CYP2R1* is the gene behind the observed GWA signal for 25(OH)D in the chromosome 11 locus [14, 16, 17, 19–21, 28].

### In the search for underlying causal variants

From the eQTL comparison analysis we observed that all analyzed SNPs could be divided into two groups. Within each group all SNPs had the same association direction over the two datasets (our study and GTEx), but the association direction was opposite between the two groups of SNPs. Looking at linkage disequilibrium (LD) patterns we observed that SNPs were in higher linkage disequilibrium within their group than with SNPs belonging to the other group. We therefore explored the idea that single underlying genetic variants are driving the observed signals. We functionally annotated all common variants (MAF≥0.05) in both loci within the 200 kb target window and focused on exonic and splice site variants in high LD (r2≥0.6) with the lead SNPs (S1 Table).

For the locus on chromosome 4, one missense variant (rs4588, p.Thr436Lys, p = 1.503^−10^, rank = 4th), in high LD with the lead SNP (r^2^ = 0.68, D’ = 0.82), was seen in *GC*. Of the 475 other variants within the target window, one other protein altering variant in *GC* was seen (rs7041, p.Asp432Glu, p = 2.252^−08^, rank = 16th), but it was in weaker LD (r2 = 0.38, D’ = 0.88) with the lead SNP. Both these variants have previously been shown to impair vitamin D binding protein function and affect 25(OH)D levels [37]. However, taking advantage of the observed LD differences, we could analyze these 2 SNPs separately by fixing the genotype for the lead SNP (rs1155563) at the major allele (T/T). In this sub-analysis neither rs7041 nor rs4588 were associated with 25(OH)D levels and thus are unlikely to be the drivers of the observed signal (S1 Appendix).

For the locus on chromosome 11, none of the 224 variants within the target window had a protein altering effect, and hence we have no obvious candidate for an underlying cause. To further explore possible candidate causal variants, we also annotated all common variants (MAF≥0.05) for the same 200 Kbp windows in both loci from publicly available whole genome sequences from 1747 Finnish individuals included in the gnomAD database [38]. Taking advantage of sequencing data made it possible to assess multiallelic sites and variants hard to impute. However, no additional variants with probable protein altering capacity were identified.

### Identifying the major haplotypes and their effects

**Fig 6:**
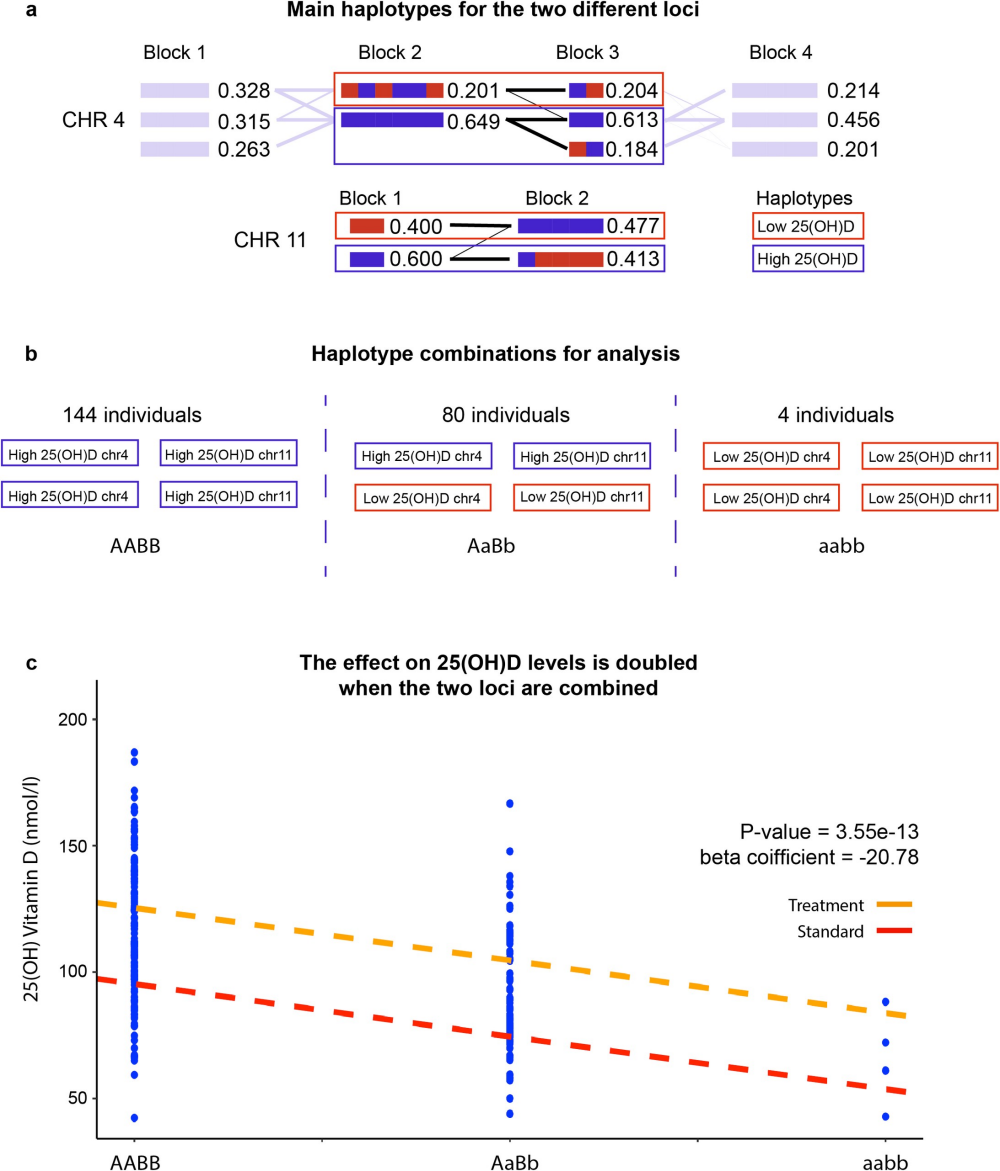
Identifying the main haplotypes for each of the two significant loci. **a)** Using the more confident non-imputed data haplotypes were constructed using Haploview for the regions surrounding both lead SNPs at each locus. The main haplotype associated with low 25(OH)D were identified for each locus. **b)** To measure the combinatory effect of ‘low 25(OH)D haplotypes’ all individuals matching a haplotype combination of AABB, AaBb or aabb were grouped together (n = 228). **c)** 25(OH)D measurement at 24 months for each individual in each haplotype group. When combining the haplotypes, the effect on the 25(OH)D concentrations is approximately doubled (beta = -20.78) compared to viewing each locus alone.

Using the non-imputed data with high genotyping frequency and the most confident genotype calls we identified the major haplotypes for the two loci using Haploview [39] [Methods]. This was an effort to capture an underlying cause without prior assumptions. Because of relatively high frequency, in both loci, of the haplotypes that included the markers associated with low 25(OH)D, we could also assess combinatory effects of these risk haplotypes under a simplified additive model. The study subjects were genetically stratified into three groups based on the individual haplotype set (Fig 6). Only individuals matching one of the three haplotype sets shown in Fig 6B were assessed in the analysis (total of 228 individuals). The results showed that the effect on 25(OH)D concentrations for the combination of risk haplotypes was additive, approximately doubling the effect. The effect on 25(OH)D concentrations was also larger than the effect seen by looking only at combinatory effects of the lead SNPs in the two loci (beta = -20.781 vs -17.736; std beta = -0.368 vs -0.323). This suggests that the haplotypes tag more of the relevant genomic information than the lead SNPs alone.

### Associating vitamin D risk haplotypes to tibial pQCT measurements

**Fig 7:**
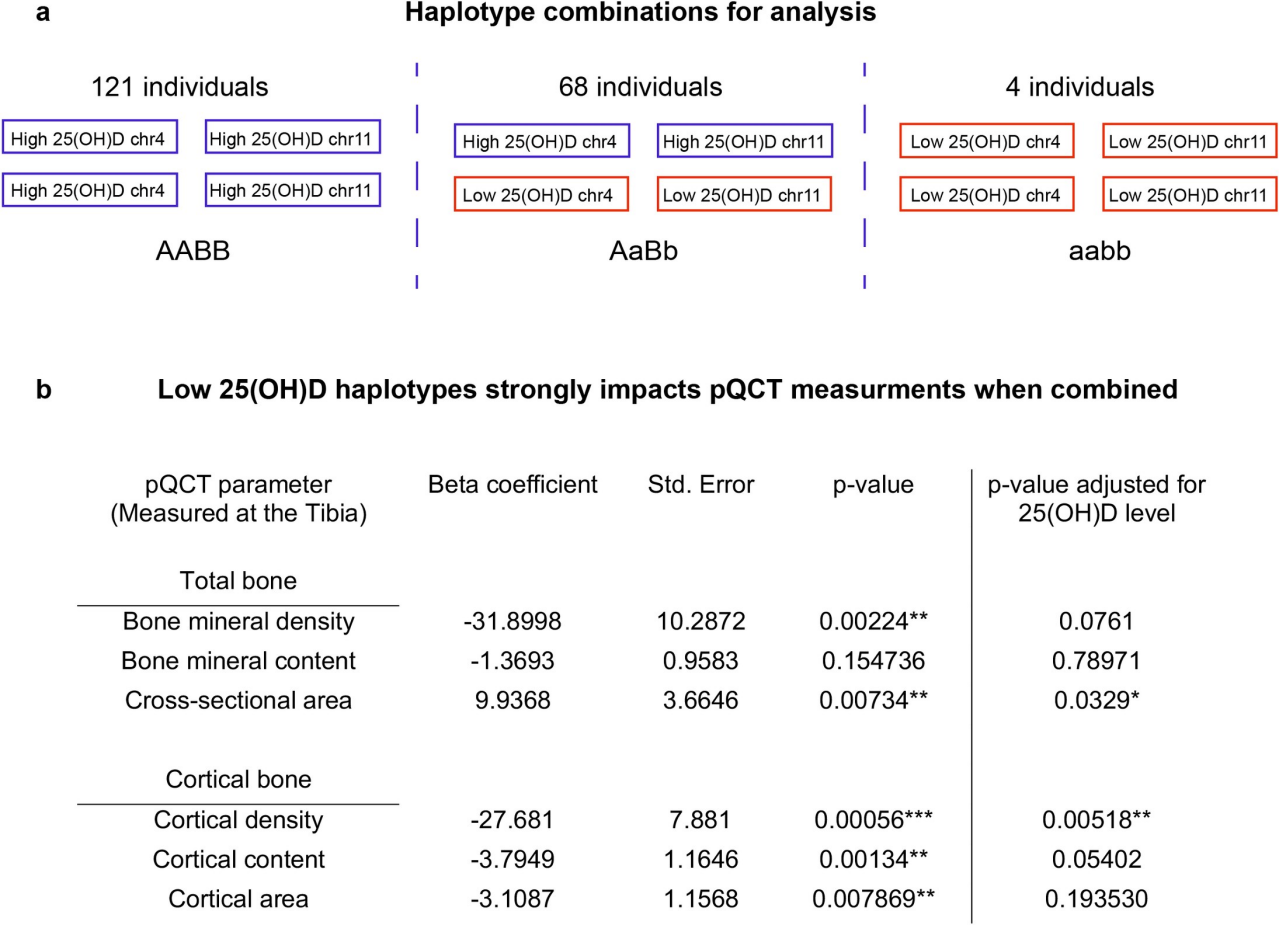
25(OH)D risk haplotypes affect skeletal parameters. **a)** The individuals are grouped based on haplotype combination (AABB, AaBb or aabb), just as in Fig 6, but here only the individuals with pQCT measurements at 24-months are shown (n = 193). **b)** When combined, the ‘low 25(OH)D’ haplotypes from the two loci are strongly associated to all pQCT-parameters except for bone mineral content (total bone). When adjusting for the 25(OH)D concentration the association disappears or weakens considerably, suggesting that vitamin D is the mediator of the effect. (** p<0.01; *** p<0.001).

We observed that 25(OH)D concentration was positively, and significantly, associated with several pQCT parameters (Table 2). Similarly, the effect alleles for both lead SNPs associated negatively with several pQCT parameters, but these associations were not statistically significant. These results suggested a relationship between 25(OH)D concentrations and pQCT parameters at 24 months. Taking advantage of the more powerful haplotype analysis (Fig 6), the results show that the haplotype combinations associating with low 25(OH)D concentrations were also strongly, and negatively, associated with several pQCT parameters (Fig 7). Comparing the association direction for ‘low 25(OH)D haplotypes’ and overall 25(OH)D concentrations, we observed the direction to be opposite for all 6 pQCT parameters (Table 2). By using this single sample Mendelian randomization approach, we can provide support for vertical pleiotropy, meaning that the concentration of 25(OH)D is not only a marker for skeletal outcomes but a mediator of the actual effect (Fig 7B). The results show that low 25(OH)D has a negative impact on bone in 24-month-old children. The results were not dependent on the 4 individuals harboring the rarest haplotype (aabb); the results remained unchanged when these individuals were omitted (S2 Table)”. The applied linear regression model was adjusted for sex, randomization group, length, length-adjusted weight and the manually assessed quality of the scan. This finding is noteworthy because most randomized clinical trials and other Mendelian randomization studies have not been able to find evidence for a causal relationship between bone mineral density and 25(OH)D [40–42]. The discordant results may relate to the young age in our study cohort, but more studies in a pediatric setting are needed to be able to draw conclusions of the age dynamics of vitamin D metabolism.

**Table 2:** Associations between serum 25(OH)D and pQCT measurements at 24 months.

pQCT parameter (Measured at the Tibia)	Beta coefficient (25(OH)D)	Std. Error	P-value	Association direction for 25(OH)D concentration		Association direction for ‘low 25(OH)D haplotypes’	
Total bone							
Bone mineral density	0.3464	0.1300	0.0079**	+	(sig.)	-	(sig.)
Bone mineral content	0.02563	0.01121	0.023*	+	(sig.)	-	(non-sig.)
Cross-sectional area	-0.08018	0.04455	0.072	-	(non-sig.)	+	(sig.)
Cortical bone							
Bone mineral density	0.18267	0.09951	0.067	+	(non-sig.)	-	(sig.)
Bone mineral content	0.03939	0.01396	0.0049**	+	(sig.)	-	(sig.)
Cross-sectional area	0.03929	0.01338	0.0034**	+	(sig.)	-	(sig.)

* p<0.05

**p<0.01

### Study limitations

We have shown that our study has several strengths, especially when it comes to the homogeneity of the study cohort and careful collection of the patient data, including skeletal outcomes [12]. This extensive collection of patient data also made it possible to interpret our findings in the context of skeletal outcomes. The intervention setting allowed us to evaluate also the effects of genetic factors on response to supplementation. However, our study also has some limitations. The cohort was underpowered in size for a genome-wide approach and can only detect genotypes that have a large effect on 25(OH)D concentrations, while loci with a more moderate effect remain undetectable. Furthermore, a replication cohort was not available to us for corroborating our results. However, despite the relatively small cohort we were able to find significant associations, further underscoring the significance of the identified genetic variants and loci. The study setting was not ideal when trying to associate genetic variation to 25(OH)D concentrations and assessing response to supplementation, because the entire cohort received vitamin D supplements in two different doses. However, due to the country’s northern location and current national vitamin D guidelines it would not be ethically possible to include a placebo group in a randomized vitamin D study in this age group. Functional experiments were not within the scope of this study and we can therefore only provide support for our findings from post hoc analyses.

### Conclusion

In this GWA study–the first of its kind in this age group—involving 761 Finnish infants we have identified two loci that strongly associate with 25(OH) Vitamin D concentrations in 24-month-old children. We also showed that the locus on chromosome 4, within the *GC* gene, seems to affect response to supplementation. We were not able to find any convincing single underlying causative variant in either locus, instead we focused on the effects seen from sets of variants within the same haplotypes. We could show that these sets of variants within the two loci not only associated with 25(OH)D concentrations but were also associated with pQCT-derived parameters of bone strength in 24-month-old children. Because the overall 25(OH)D concentration also was associated with pQCT-derived parameters in a coherent manner, the results suggest that the haplotypes’ effect on bone are, at least partly, mediated by 25(OH)D.

## Methods

### Ethics statement

The study was approved by the Research Ethics Committee of the Hospital District of Helsinki and Uusimaa (permit id: 107/13/03/03/2012) and the trial protocol is registered in ClinicalTrials.gov (NCT01723852). All parents gave their written informed consent at recruitment.”

### Subjects and study setting

In this study we used genetic data for altogether 928 children participating the Vitamin D intervention (VIDI) study. The VIDI study, described more in detail by Rosendahl et al [12, 43], was a randomized clinical trial including 975 new-born infants who were recruited between January 2013 to June 2014 at Kätilöopisto Helsinki Maternity Hospital, Helsinki, Finland. All children were born at term with a normal birth weight to healthy mothers of Northern European descent [44]. The VIDI study investigated whether a daily dose of 30 μg (1200 IU) of Vitamin D_3_, instead of the standard daily dose of 10 μg (400 IU), had an impact on tibial strength or parent-reported infections at 24 months. The children were randomized in 1:1 ratio to receive the lower (Group_10_) or higher (Group_30_) supplemental dose from age 2 weeks to 24 months. As previously reported [12], we saw no difference between the two groups for the two primary endpoints: (1) tibial strength or (2) parent-reported infections at 24 months.

During the 24-month intervention, birth medical records where evaluate and the children had follow-up visits at 6, 12 and 24 months. The anthropometric measurements included length, weight, length-adjusted weight (in %) and head circumference. 25(OH)D was measured from the blood samples taken from the umbilical cord, serum 25(OH)D was assessed at 12 and 24 months and skeletal measurements were obtained at 12 and 24 months using peripheral quantitative computed tomography (pQCT, Stratec XCT2000LResearch+; Stratec Medizintechnik GmbH) measured at the 20% distal site of the left tibia [12]. Our analyses focused on three pQCT parameters (bone mineral density, bone mineral content and cross-sectional area) for both total and cortical bone at the 24-month time point. The mothers’ 25(OH)D concentration was measured during pregnancy at their regular follow-up visit, on average at gestational week 11 (data for 648 mothers were available to us) [13].

### Genotyping of the cohort

DNA was extracted from umbilical cord blood samples in the laboratory of the Finnish National Institute for Health and Welfare, using automated Chemagen MSM1 extraction (PerkinElmer Inc., Chemagen 140 Technologie GmbH, Baesweiler, Germany) or the Gentra Puregene—kit (Qiagen GmgH, Hilden, Germany). DNA was available for 928 VIDI participants and the samples were sent simultaneously for genotyping using the Illumina Infinium Global Screening Array v1.0 at the Human Genomics Facility (HuGe-F) at Erasmus MC, Netherlands. The raw data included a total of 686,085 different genotyped variants across the genome.

#### Quality control

We excluded 7 samples (0.75%) because of poor genotyping quality (>5% missing genotypes) and 1 sample because of a mismatch in the computationally inferred sex and the reported clinical sex. Kinship coefficients were calculated using the KING software [45], the cutoff was set to <0.177, aiming to exclude duplicate samples and first degree relatives, but no samples failed to pass this criterion. Overall the genotype quality was excellent with a total genotyping rate of 0.9986.

#### Ancestry mapping

All samples were collected in Helsinki, Finland from children born to mothers of Northern European decent, the vast majority being of entirely Finnish descent. To ensure homogeneity regarding population structure, we performed ancestry inference using the TRACE software, which is contained within the LASER suite [46, 47]. A 4-dimension reference space was created using the European subset of the 1000 genomes data phase 3, in total 503 individuals, of which 99 individuals are of Finnish descent. We created a 20-dimension genetic map by applying a principal component analysis on each individual together with the reference population. All individuals were then projected from their 20-dimensional map in to the 4-dimensional reference space (S3 Fig). We excluded 22 samples because they were deemed to be genetically too far from the Finnish reference individuals. For a detailed workflow description of the data processing performed before and during the association test please see S2 Appendix.

#### Imputation

We performed imputation, using the 1000 genomes phase 3 data as reference, on all samples that passed the filtering criteria. No filtering of the data was performed prior to imputation, a strategy supported by Roshyara et al. [48] Genotypes were first phased using SHAPEIT (version 2) [49], reference alleles were updated and strand issues were resolved using Genotype harmonizer [50]. IMPUTE2 was then used for genotype imputation [51, 52]. We performed post imputation filtering and excluded SNPs not fulfilling the following 5 criteria; (1) biallelic SNP, (2) IMPUTE2 info score ≥0.8, (3) minor allele frequency ≥0.05, (4) missing genotype calls ≤5% (5) Hardy Weinberg equilibrium p≥0.00001. A genotype with an info score ≥0.4 is usually considered well imputed, but because of our small sample size we chose a more conservative threshold (info score ≥0.8), to limit the analysis to confident genotype calls. A Hardy Weinberg equilibrium (HWE) cutoff of p≥0.00001 was chosen after assessing QQ-plot of the distribution of the calculated HWE p-values (S4 Fig). The final number of SNPs eligible for the association test was 5,072,729. The genetic regions within 2.5 Mb from the lead SNP in each locus were re-imputed using the standard algorithm from IMPUTE2 on the non-phased genotypes, which should yield slightly more precise results. During the re-imputation process we also chose to keep small indels.

### Genome-wide association test on 25(OH)D concentrations

We carried out the genome-wide association test using the 25(OH)D concentration at 24 months as a continuous variable in a linear regression analysis using the software Plink (v 1.9)[53]. The analysis was restricted to the 761 children with 25(OH)D measurement at 24 months. Covariables used in the model were: 1) sex; 2) randomization group, either the standard dose of 10 μg (Group_10_) or the higher dose of 30 μg (Group_30_) of daily vitamin D; 3) season for 25(OH)D measurement; 4) the first 4 principal components. FlashPCA was used to conduct the principal component analysis [54].

### eQTL analysis (GTEx data)

We compared our genome-wide association results with publicly available eQTL data from the GTEx project (V7). Summary data for association results between genetic variation and gene expression was downloaded from the GTEx portal (gtexportal.org/home) for the tissues “Stomach”, “Thyroid”, “Pancreas”and “Nerve-Tibial”. In these 4 tissues at least one of our top associated SNPs, either on chromosome 4 or chromosome 11, also showed a significant association to gene expression levels in the GTEx dataset. Data for the genetic regions within 100 kb of the lead SNP in each locus were extracted for the above tissues. The sequential analysis comparing our association data to the GTEx eQTL data was limited to the two 200 kb target windows, and therefore, after intersecting the two datasets, we also re-adjusted the significance threshold to a false discovery rate ≤0.05. The probability for shared underlying genetics for SNPs associated to 25(OH)D and GTEx gene expression level was estimated for all intersecting SNPs within the target window for each locus using an approximate bayes factor colocalization analysis. To perform the analysis the R package “coloc” was used [32]. All data processing and graphics were created in R (version 3.3.3).

### Annotation of functional variants

We investigated the possibility of identifying single underlying variants responsible for the association signals seen on chromosome 4 and chromone 11. We annotated 100 kb regions on each side of the lead SNP in both loci using variant effect predictor (VEP) [55]. Indels were normalized using Vt [56] and strand harmonization against the GRCh37 reference sequence was performed using BEDTools[57] and Plink. Possibly functional variants (exonic and splice site variants) were extracted using GEMINI [58]. We also downloaded summary data from whole genome sequences of 1747 Finnish individuals contained within the gnomAD database (gnomad.broadinstitute.org). All variants recorded from the 1747 Finnish individuals within the two target windows were analyzed in the same manner as above.

### Haplotype analysis

We identified the major haplotypes for the observed GWA loci on chromosome 4 and 11 using Haploview[39]. Based on the Haploview analysis, three non-imputed markers per locus were selected to discriminate between the major haplotypes (frequency ≥10%) at each locus. All three non-imputed markers were genome wide significant, in high LD, and with a very high genotype call rate (Fig 6A). The markers selected were rs1155563, rs2282679, rs17467825 for the locus on chromosome 4, and rs11023350, rs1007392, rs11023332 for the locus on chromosome 11. The haplotype associated with low 25(OH)D on chromosome 4 and chromosome 11 were denoted as a and b respectively, while the haplotypes associated with high 25(OD)D were denoted as A and B. To assess the combinatory effects of the major haplotypes at the two loci we created a simplified additive linear model where we genetically stratified the study subjects to three groups based on the individual haplotype set (AABB, AaBb and aabb). Hence, the individuals included in the analysis had to have: (1) Two haplotypes associated with high 25(OH)D at both loci (AABB), or (2) Two haplotypes associated with low 25(OH)D at both loci (aabb), or (3) One haplotype associated with high 25(OH)D and one haplotype associated with low 25(OH)D at both loci (AaBb) (Fig 6). In total 228 individuals matched one of these 3 haplotype combination and were eligible for the analysis. This allowed us to calculate the combinatory effects of the haplotypes on 25(OH)D concentrations. The linear model was, just as in the genome-wide association test, adjusted for sex, season for 25(OH)D measurement and randomization group.

The same genetically stratified groups (individuals with a haplotype set of: AABB, AaBb or aabb) were compared against all 6 pQCT parameters using the same linear model, but now adjusted for sex, randomization group, length (cm), length to weight ratio (SD) and manually assessed quality of the scan [12]. Using dual outcome variables on the same genetically stratified group allowed us to assess pleiotropy. Altogether 228 children were eligible for the haplotype analysis, having a 25(OH)D measurement at 24-month and matching a haplotype combination of either AABB, AaBb or aabb. Of these, 193 children also had pQCT measurements at 24 months (Fig 7).

### Statistical analyses

We used the standard genome wide significance threshold of 5x10^-8^ in our association analysis, which can be considered appropriate since we only included common SNPs (MAF≥0.05) in the analysis. Missing values for the different parameters are presented for each analysis. Plink (v1.9) was used for the genome wide association test, while all other statistical models and plots were created in R (version 3.3.3). R packages used were: “car”, “coloc”, “ggplot2”, “lm.beta”, “lme4” and “qqman”. Assumptions for all linear models have been checked for normality, equal variance and linearity.

## Supporting information

S1 Fig QQ-plot for the association test. As we can see from the qq-plot we see more SNPs strongly associated to 25(OH)D levels than expected by chance. We have a genomic inflation factor (λ) of 0.997, suggesting that the associations we see are true and not due to bad quality data or population stratification.
(TIF)

S2 Fig Study data vs GTEx data for the chromosome 11 locus. Comparison of the association statistics for 25(OH)D in 24-month old children within the 200kb target window on chromosome 11 compared to the GTEx eQTL dataset for the genes *PDE3B* (tissue: Pancreas) and *RRAS2* (tissue: Tibial Nerve). All SNPs that both show a significant association statistic and are present in both datasets are colored either blue (-) or red (+), depending on the direction of the SNP’s beta coefficient. The results show, just as with *CYP2R1* (tissue: Thyroid) a complete concordance in the direction of SNPs’ association with 25(OH)D levels in our study and gene expression in the GTEx dataset. Our study data is presented above the line y = 0 and the GTEx data below it. (red line: genome-wide significance; blue line: genome-wide suggestive significance; orange line: FDR 0.05)
(TIF)

S3 Fig Ancestry inference. A 4-dimensional reference space was created using data from the European subset of the 1000 genomes phase 3 population. Out of the 503 European individuals, 99 have Finnish descent. Each study individual has subsequently been projected from a 20-dimensional genetic map onto the 4-dimensional reference map. To the left the study individuals have been overlaid (black dots), to the right the original reference maps are shown. The black rectangle describes the space within 4SD of the 99 Finnish individuals.
(TIF)

S4 Fig QQ-plot of HWE p-values. When using a Hardy Weinberg equilibrium p-value cutoff of 0.00001 we see that we that the genotype counts does not deviate from HWE equilibrium more than expected.
(TIF)

S1 Table Variants in high LD (r^2^>0.6) with the lead SNP within 100 kb from the lead SNP in each locus. (DOCX)

S2 Table Low 25(OH)D haplotypes associates with pQCT parameters also when the 4 individuals matching the rarest haplotype (aabb) are excluded. (DOCX)

S1 Appendix Supporting information. (DOCX)

S2 Appendix Complete workflow. (DOCX)
