# Supplementary figures and images for: Genetic variation in GC and CYP2R1 affects 25-hydroxyvitamin D concentration and skeletal parameters: A genome-wide association study in 24-month-old Finnish children
Source: PLoS Genet. 2019 Dec 16;15(12):e1008530. doi: 10.1371/journal.pgen.1008530 (PMC6936875; doi:10.1371/journal.pgen.1008530)

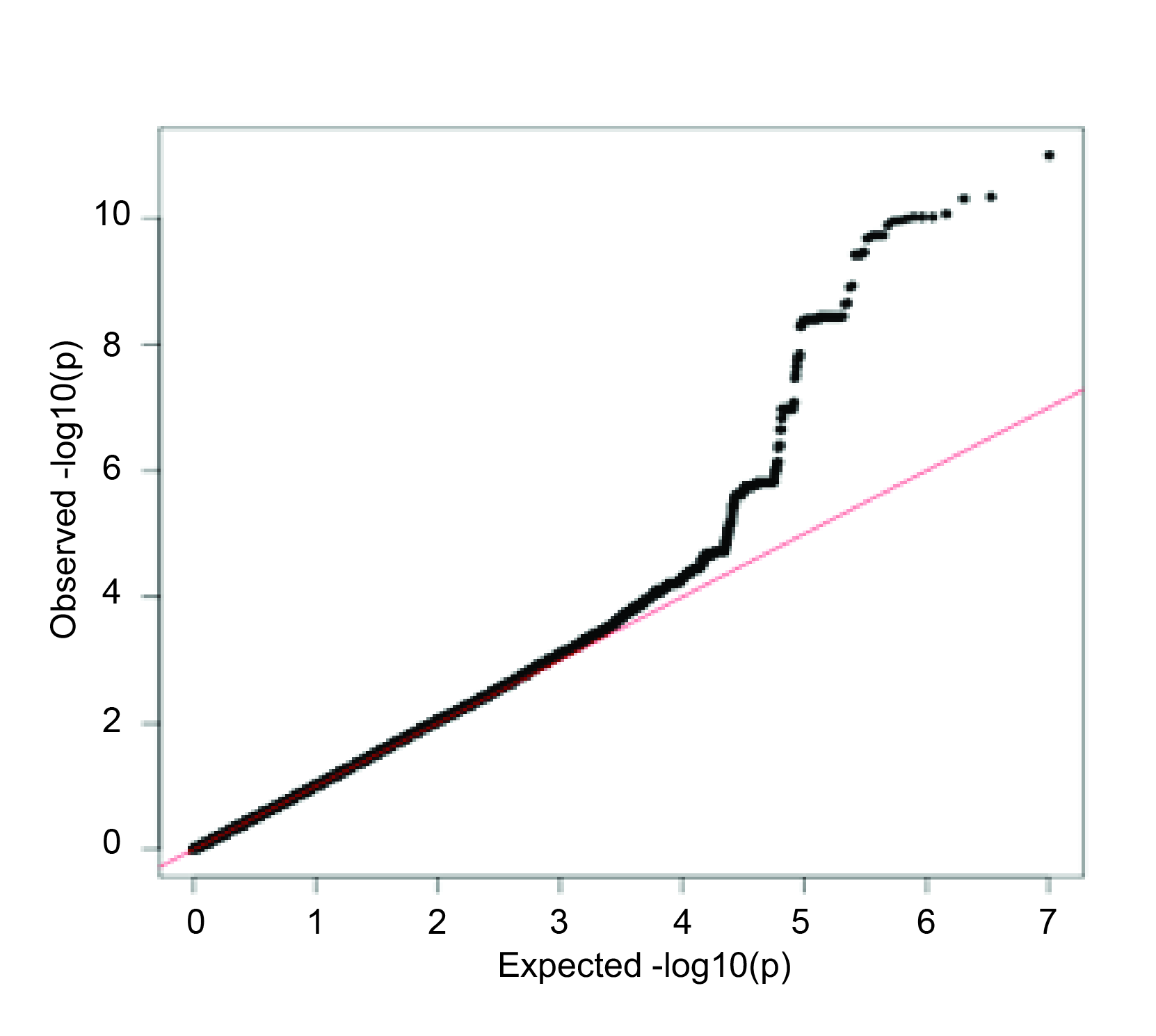

Supplement: S1 Fig — As we can see from the qq-plot we see more SNPs strongly associated to 25(OH)D levels than expected by chance. We have a genomic inflation factor (λ) of 0.997, suggesting that the associations we see are true and not due to bad quality data or population stratification. (TIF) [file pgen.1008530.s001.tif]

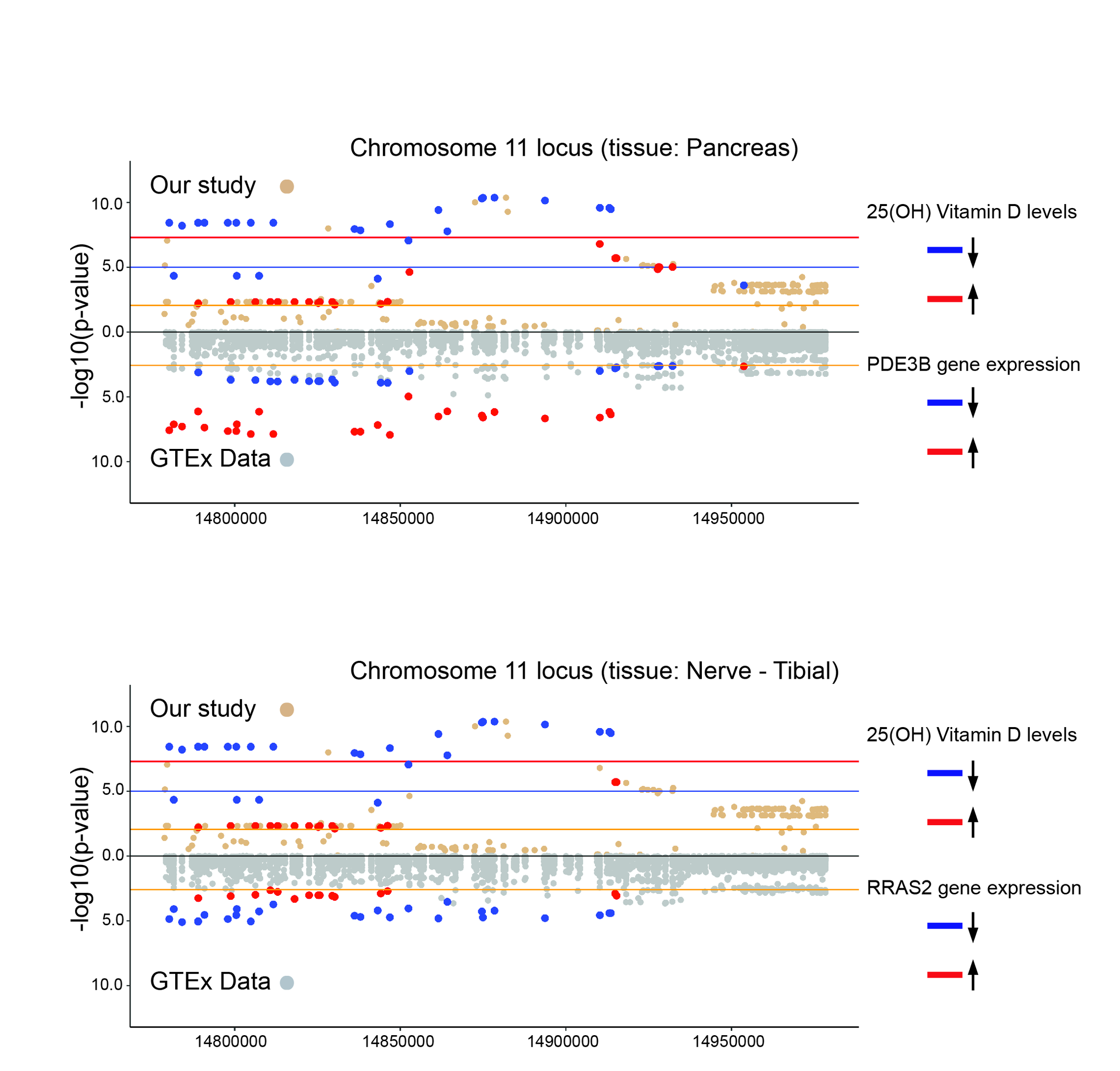

Supplement: S2 Fig — Comparison of the association statistics for 25(OH)D in 24-month old children within the 200kb target window on chromosome 11 compared to the GTEx eQTL dataset for the genes PDE3B (tissue: Pancreas) and RRAS2 (tissue: Tibial Nerve). All SNPs that both show a significant association statistic and are present in both datasets are colored either blue (-) or red (+), depending on the direction of the SNP’s beta coefficient. The results show, just as with CYP2R1 (tissue: Thyroid) a complete concordance in the direction of SNPs’ association with 25(OH)D levels in our study and gene expression in the GTEx dataset. Our study data is presented above the line y = 0 and the GTEx data below it. (red line: genome-wide significance; blue line: genome-wide suggestive significance; orange line: FDR 0.05) (TIF) [file pgen.1008530.s002.tif]

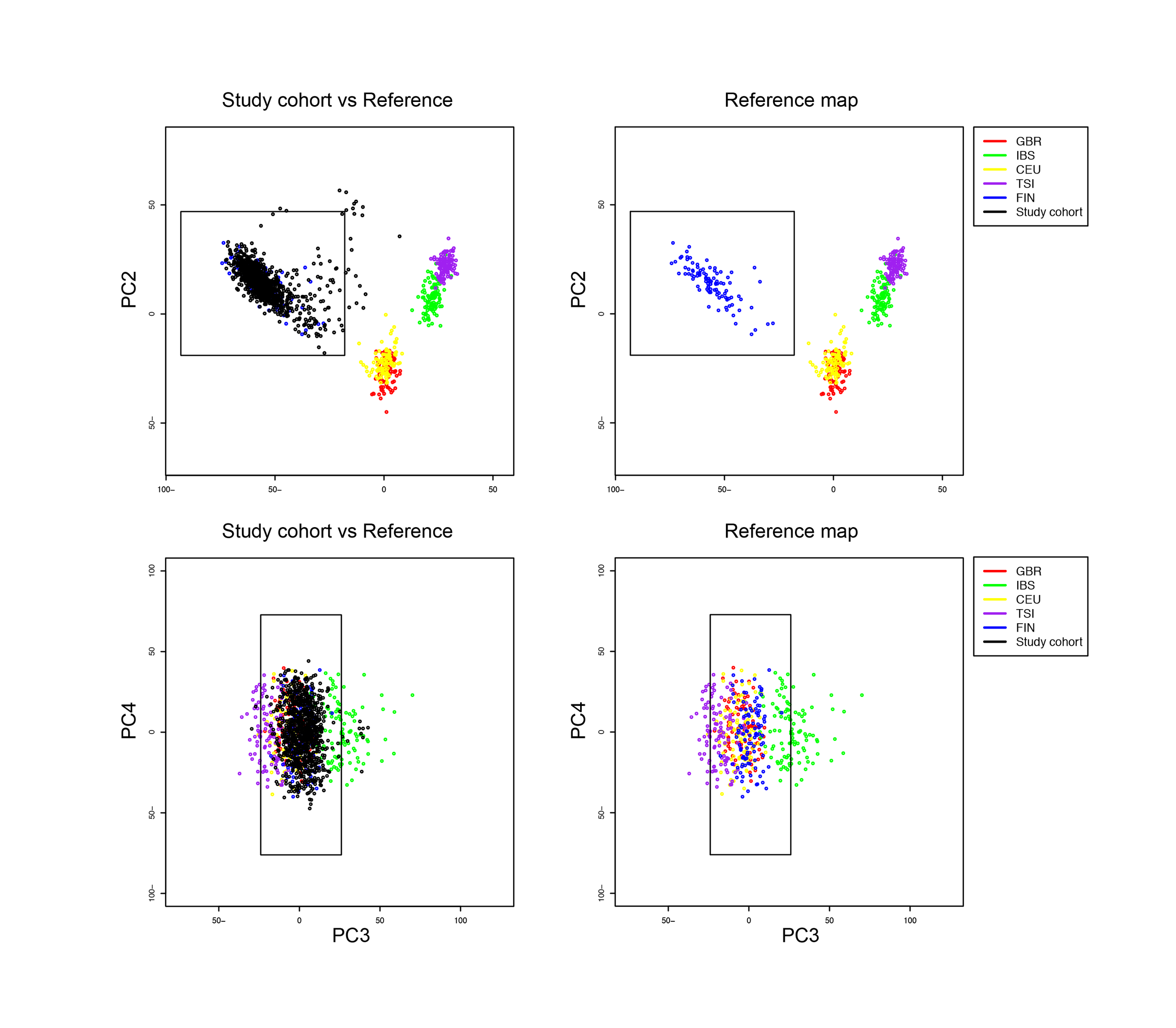

Supplement: S3 Fig — A 4-dimensional reference space was created using data from the European subset of the 1000 genomes phase 3 population. Out of the 503 European individuals, 99 have Finnish descent. Each study individual has subsequently been projected from a 20-dimensional genetic map onto the 4-dimensional reference map. To the left the study individuals have been overlaid (black dots), to the right the original reference maps are shown. The black rectangle describes the space within 4SD of the 99 Finnish individuals. (TIF) [file pgen.1008530.s003.tif]

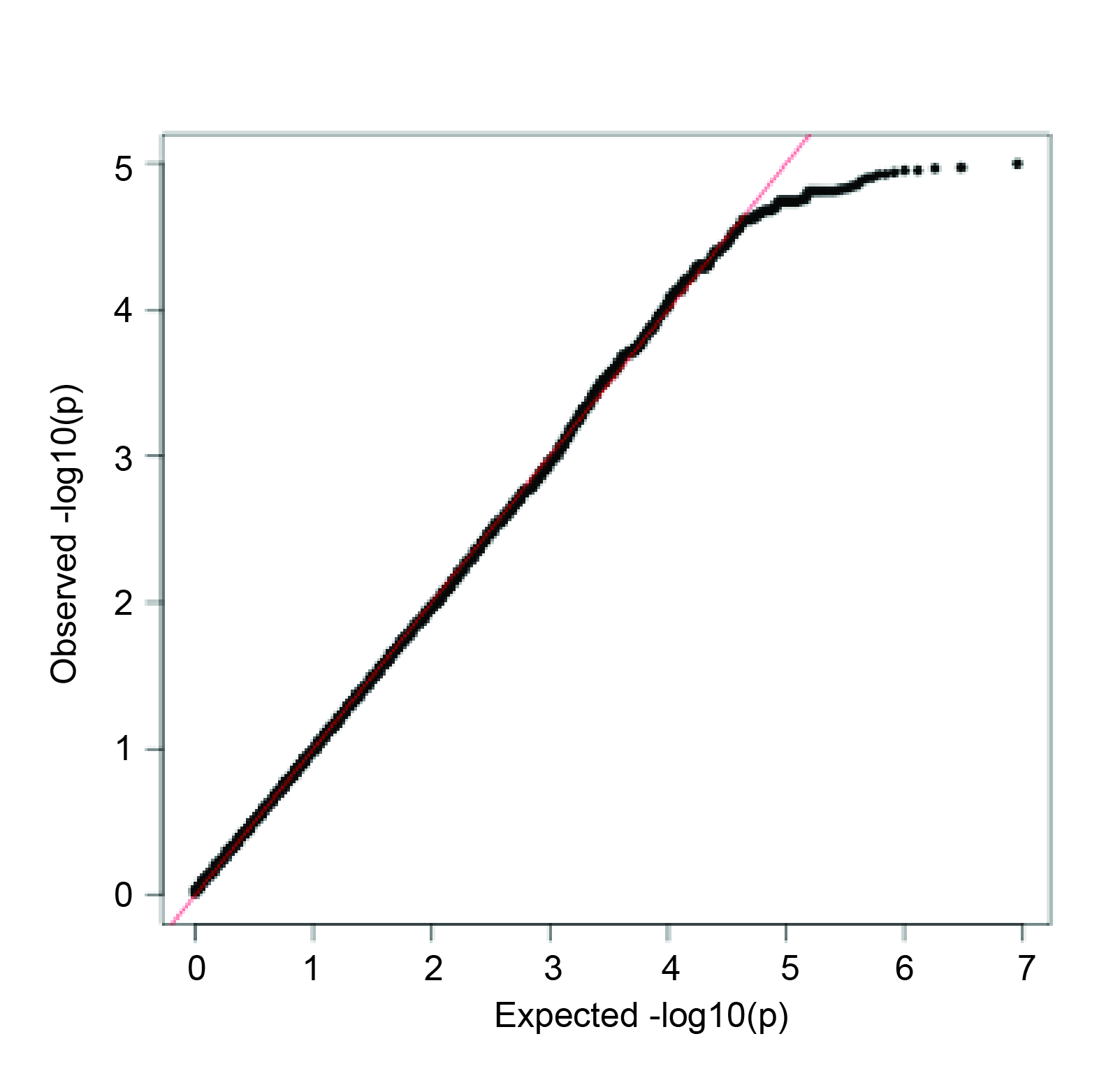

Supplement: S4 Fig — When using a Hardy Weinberg equilibrium p-value cutoff of 0.00001 we see that we that the genotype counts does not deviate from HWE equilibrium more than expected. (TIF) [file pgen.1008530.s004.tif]
