# Supplementary material for: Genetic variation in GC and CYP2R1 affects 25-hydroxyvitamin D concentration and skeletal parameters: A genome-wide association study in 24-month-old Finnish children
Source: PLoS Genet. 2019 Dec 16;15(12):e1008530. doi: 10.1371/journal.pgen.1008530 (PMC6936875; doi:10.1371/journal.pgen.1008530)
Supplement: S1 Table — (DOCX) [file pgen.1008530.s005.docx]

**Chromosome 4 locus**

| **SNP** | **NMISS** | **P** | **impact** | **gene** | **LD(r^2^)** | **D-prime** | **MAF** |
| --- | --- | --- | --- | --- | --- | --- | --- |
| rs1155563 | 761 | 1.011e-11 | intron variant | GC | 1.00 | 1 | 0.2041 |
| rs62302167 | 742 | 7.268e-11 | intron variant | GC | 0.994362 | 1 | 0.200727 |
| 4:72623324  A:AATGAC | 743 | 6.865e-11 | intron variant | GC | 0.726775 | 0.856165 | 0.20082 |
| rs4588 | 757 | 1.503e-10 | missense variant | GC | 0.675523 | 0.82304 | 0.202679 |
| rs34265662 | 757 | 1.503e-10 | intron variant | GC | 0.674976 | 0.822709 | 0.202413 |
| rs1352846 | 757 | 1.503e-10 | intron variant | GC | 0.674976 | 0.822709 | 0.202413 |
| rs11723621 | 749 | 1.509e-10 | intron variant | GC | 0.656631 | 0.815 | 0.195475 |
| rs368486133 | 752 | 1.854e-10 | intron variant | GC | 0.64594 | 0.807159 | 0.196123 |
| rs3755967 | 753 | 1.948e-10 | intron variant | GC | 0.641781 | 0.806848 | 0.19577 |
| rs2282680 | 761 | 1.859e-10 | intron variant | GC | 0.6143 | 0.805942 | 0.194938 |
| rs17467825 | 760 | 1.809e-10 | downstream gene variant | GC | 0.61412 | 0.805834 | 0.195285 |
| rs2282679 | 761 | 1.859e-10 | Intron variant | GC | 0.611751 | 0.805412 | 0.194494 |

**Chromosome 11 locus**

| **SNP** | **NMISS** | **P** | **impact** | **gene** | **LD (r^2^)** | **D-prime** | **MAF** |
| --- | --- | --- | --- | --- | --- | --- | --- |
| rs10832310 | 755 | 4.241e-11 | intron variant | PDE3B | 1.00 | 1.00 | 0.373201 |
| rs10832311 | 755 | 4.241e-11 | intron variant | PDE3B | 1.00 | 1.00 | 0.373201 |
| rs731042 | 756 | 4.303e-11 | intron variant | PDE3B | 1.00 | 1.00 | 0.373429 |
| rs34555721 | 755 | 5.013e-11 | intron variant | PDE3B | 1.00 | 1.00 | 0.373765 |
| rs71044027 | 754 | 7.105e-11 | downstream gene variant | PDE3B | 1.00 | 1.00 | 0.373537 |
| rs11023364 | 757 | 9.764e-11 | intron variant | PDE3B | 1.00 | 1.00 | 0.374104 |
| rs10500804 | 747 | 2.56e-10 | intron variant | CYP2R1 | 1.00 | 1.00 | 0.376937 |
| rs10766196 | 746 | 2.612e-10 | 5-prime UTR variant | CYP2R1 | 1.00 | 1.00 | 0.377514 |
| rs12794714 | 746 | 3.288e-10 | synonymous variant | CYP2R1 | 1.00 | 1.00 | 0.377626 |
| rs201501563 | 728 | 5.147e-10 | intron variant | PDE3B | 1.00 | 1.00 | 0.360671 |
| rs11023350 | 761 | 3.797e-10 | intron variant | PDE3B | 0.946139 | 1.00 | 0.388099 |
| rs12795794 | 753 | 4.674e-09 | intron variant | PDE3B | 0.927596 | 1.00 | 0.390583 |
| rs75828815 | 761 | 3.67e-09 | intron variant | PDE3B | 0.911512 | 0.989928 | 0.391652 |
| rs10832300 | 761 | 3.67e-09 | intron variant | PDE3B | 0.911512 | 0.989928 | 0.391652 |
| rs10832301 | 761 | 3.67e-09 | intron variant | PDE3B | 0.911512 | 0.989928 | 0.391652 |
| rs12416696 | 761 | 3.67e-09 | intron variant | PDE3B | 0.911512 | 0.989928 | 0.391652 |
| rs3206554 | 761 | 3.67e-09 | intron variant | PDE3B | 0.911512 | 0.989928 | 0.391652 |
| rs10047458 | 761 | 3.67e-09 | intron variant | PDE3B | 0.911512 | 0.989928 | 0.391652 |
| rs76739648 | 761 | 3.67e-09 | intron variant | PDE3B | 0.911512 | 0.989928 | 0.391652 |
| rs10766192 | 761 | 3.67e-09 | intron variant | PDE3B | 0.911512 | 0.989928 | 0.391652 |
| rs10832303 | 757 | 1.001e-08 | intron variant | PDE3B | 0.911001 | 0.989864 | 0.390277 |
| rs11023332 | 757 | 6.203e-09 | intron variant | PDE3B | 0.909357 | 0.989865 | 0.392857 |
| rs4373971 | 744 | 8.802e-08 | intron variant | PDE3B | 0.886351 | 0.966894 | 0.377727 |
| rs34041254 | 756 | 1.399e-08 | intron variant | PDE3B | 0.870941 | 0.993724 | 0.404103 |
| rs4757269 | 757 | 1.121e-08 | intron variant | PDE3B | 0.869417 | 0.993723 | 0.404635 |
| rs10766194 | 736 | 1.682e-08 | intron variant | PDE3B | 0.678958 | 1.00 | 0.474288 |
| rs1037378 | 760 | 8.756e-08 | intron variant | PDE3B | 0.642231 | 1.00 | 0.481713 |
| rs1451678 | 760 | 4.576e-05 | intron variant | PDE3B | 0.638937 | 0.987163 | 0.477313 |
| rs4281505 | 760 | 4.576e-05 | intron variant | PDE3B | 0.638937 | 0.987163 | 0.477313 |
| rs2122942 | 760 | 4.576e-05 | intron variant | PDE3B | 0.638937 | 0.987163 | 0.477313 |
| rs78285608 | 752 | 7.612e-05 | intron variant | PDE3B | 0.620554 | 1.00 | 0.489677 |
| rs36026864 | 750 | 5.549e-06 | intron variant | CALCB | 0.60515 | 0.991813 | 0.498645 |
