# Supplementary material for: Genetic variation in GC and CYP2R1 affects 25-hydroxyvitamin D concentration and skeletal parameters: A genome-wide association study in 24-month-old Finnish children
Source: PLoS Genet. 2019 Dec 16;15(12):e1008530. doi: 10.1371/journal.pgen.1008530 (PMC6936875; doi:10.1371/journal.pgen.1008530)
Supplement: S2 Table — (DOCX) [file pgen.1008530.s006.docx]

| pQCT parameter  (Measured at the Tibia) | Beta coefficient | Std. Error | p-value | p-value adjusted for 25(OH)D |
| --- | --- | --- | --- | --- |
|  |  |  |  |  |
| Total bone |  |  |  |  |
| Total bone density | -32.9420 | 11.4292 | 0.00443** | 0.0877 |
| Total bone content | 0.9450 | 1.0593 | 0.37354 | 0.89465 |
| Total bone area | 12.2252 | 4.0506 | 0.00292** | 0.0133* |
|  |  |  |  |  |
| Cortical bone |  |  |  |  |
| Cortical density | -31.360 | 8.732 | 0.000425*** | 0.00316** |
| Cortical content | -4.0814 | 1.2925 | 0.00187** | 0.04977* |
| Cortical area | -3.2944 | 1.2843 | 0.01114* | 0.190940 |

**Low 25(OH)D haplotypes associates with pQCT parameters also when the 4 individuals matching the most rare haplotype (aabb) are excluded.**
