# Supplementary material for: Genetic variation in GC and CYP2R1 affects 25-hydroxyvitamin D concentration and skeletal parameters: A genome-wide association study in 24-month-old Finnish children
Source: PLoS Genet. 2019 Dec 16;15(12):e1008530. doi: 10.1371/journal.pgen.1008530 (PMC6936875; doi:10.1371/journal.pgen.1008530)
Supplement: S1 Appendix — (DOCX) [file pgen.1008530.s007.docx]

S1 Appendix

**Title: Genetic variation in *GC* and *CYP2R1* affects 25(OH)D levels and skeletal parameters: A genome wide association study in 24-month-old Finnish children.**

Kämpe AJ MD ^1,2^, Enlund-Cerullo M MD ^3,4,5^, Valkama S MD ^3,5^, Holmlund-Suila E ^3^, MD PhD, Rosendahl J ^3,5^ MD PhD, Hauta-alus H MSc ^3,5^, Pekkinen M PhD ^3,4,5^, Andersson S MD PhD ^3^, Mäkitie O MD PhD^1,2,3,4,5^

Affiliations

^1^ Department of Molecular Medicine and Surgery, Karolinska Institutet, Stockholm, Sweden.

^2^ Department of Clinical Genetics, Karolinska University Hospital, Stockholm, Sweden

^3^ Children’s Hospital, Pediatric Research Center, University of Helsinki and Helsinki University Hospital, Helsinki, Finland

^4^ Folkhälsan Research Center, Helsinki, Finland

^5^ Research Program for Clinical and Molecular Metabolism, Faculty of Medicine, University of Helsinki, Finland

**Index**

**Results and Discussion**

Section 1. Treatment response …………………………………………… 2

Section 2. Functional SNPs at the *GC* locus ………….………………….……………. 5

Section 3. Colocalization analysis ………….………………….……………. 6

**Section 1. Response to supplementation**

A random intercept mixed linear model was constructed to assess if the lead SNPs in each locus, depending on genotype, had different effects depending on the supplementation dose. 25(OH)D concentrations at birth and at 24 months was used as outcome and the intervention group / non-intervention group was analyzed separately. The interaction effect between genotype and time as assessed. The results were significant only in the intervention group when assessing the interaction with time for the genotype of the lead SNP on chromone 4. The results support that the individuals genotype for the lead SNP on chromosome 4 (rs1155563) can modulate the effect of supplementation.

**Random intercept mixed linear model**

The lmer function in the R package lme4 was used to build the model.

Covariates used: Gender and the mothers 25(OH)D level during pregnancy.

*Lead SNP at chromosome 4*

*Intervention group:*

*Fit1 <- lmer(25(OH)D ~ genotype*timepoint + Gender + 25(OH)D_mother + (1|ID), data=intervention group, REML=FALSE)*

*fit2 <- lmer(25(OH)D ~ genotype+timepoint + Gender + 25(OH)D_mother + (1|ID), data=intervention group, REML=FALSE)*

*anova(fit1,fit2)*

*Df AIC BIC logLik deviance Chisq Chi Df Pr(>Chisq)*

*fit2 7 5546.9 5577.8 -2766.5 5532.9*

*fit 8 5544.1 5579.4 -2764.1 5528.1 4.8292 1* ***0.02798 ****

*Interaction effect:* genotype:time: -7.40596

For the non-intervention group the interaction between genotype and time where non-significant (p=0.2428, Interaction effect: genotype:time: -3.60642), but had the same association direction. This result is reasonable since the non-intervention group received Vitamin D supplements in a lower dose (10µg/day), which should lead to a smaller interaction effect. In this case the interaction effect is too small to be significant, but this could be due to a lack of power.

**Figure: S1** Appendix. Plot of 25(OH)D concentrations related to genotype and time point for the lead SNP on chromosome 4 (rs1155563).

**
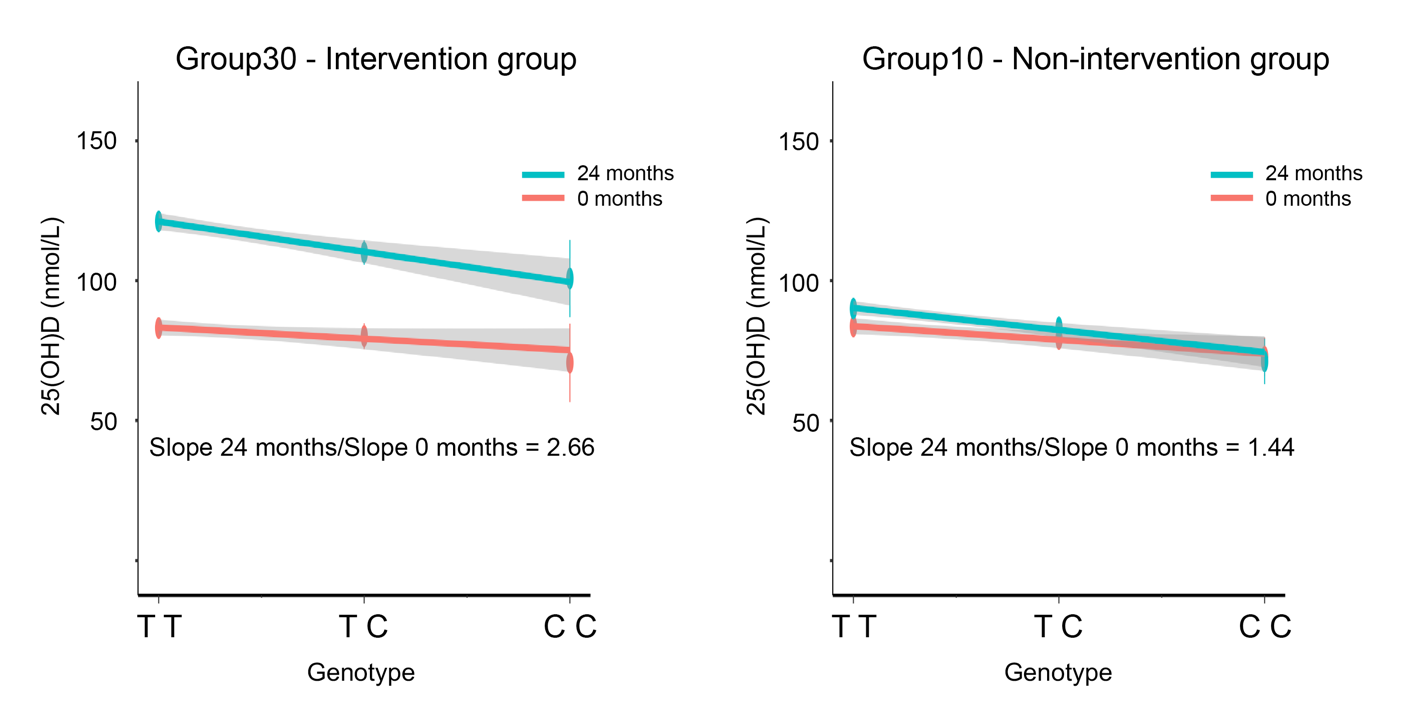
**

***In an attempt to visualize the effect on the genotype at the locus on chromosome 4 the 25(OH)D concentration at the two different time points have been plotted against the genotype for both the intervention group and the non-intervention group. The figures show that the effect size (slope) on 25(OH)D concentrations for the genotype is larger at the 24-month time point in the intervention group, as indicated by the slope ratios (2.66 vs 1.44).***

**Chromosome 11 locus**

The results for the lead SNP in the chromosome 11 locus, when performing the same analysis, does not show any significant interaction effects for any of the groups (p=0.36 (intervention group), p= 0.20 (non-intervention group)).

**Section 2. Functional SNPs at the *GC* locus**

**Separating the effects of rs1155563, rs7041 and rs4588.** To separate the effectof the lead SNP (rs1155563) and the two possibly functional SNPs (rs7041 and rs4588**)** we took advantage of the observed LD differences. The SNPs rs7041 and rs4588 were separately analyzed by fixing the genotype for the lead SNP (rs1155563) at the major allele (T/T). In this new subgroup of 482 children the genotypes for rs7041 were; 294 (C/C), 174 (A/C), 14 (A/A) and for rs4588; 439 (G/G), 42 (T/G), 0 (T/T), but now neither rs7041 nor rs4588 were significantly associated with 25(OH)D levels (p=0.551 and p= 0.236). When instead fixing the genotype for rs4588 at the major allele (G/G), the genotypes for rs7041 were; 306 (C/C), 154 (A/C), 13 (A/A) and for rs1155563; 439 (T/T), 34(C/T), 0 (C/C). In this second sub-analysis rs7041 was still not associated with 25(OH)D levels (p=0.448), but rs1155563 was (beta= -8.97, p=0.0258). The effect size for rs1155563 was similar to rs1155563’s effect size in the entire cohort (beta= -9.49). Together these sub-analyses strongly suggest that neither rs7041 nor rs4588 are the drivers of the signal on chromosome 4 in our cohort, despite the fact that they previously have been reported as functional.

**Colocalization analyses**

To quantify the association similarities seen in the eQTL comparison analysis (Fig 5) for the target windows in each locus, we used an approximate bayes factor colocalization analysis implemented in the R package “coloc” [1]. The posterior probability for 5 scenarios were calculated for each pairwise comparison between SNPs associated to 25(OH)D in our dataset and to gene expression (one tissue, one gene) in the GTEx dataset.

**The 5 scenarios:**

𝐻0: neither trait has a genetic association in the region

𝐻1: only trait 1 has a genetic association in the region

𝐻2: only trait 2 has a genetic association in the region

𝐻3: both traits are associated, but with different causal variants

𝐻4: both traits are associated and share a single causal variant

**Chromosome 4 locus (200 kb target window).**

**Comparison: Our data (25(OH)D) vs *GC* gene expression**

Probability for each scenario (%)

nsnps 91 (n)

PP.H0.abf 7.8e-06%

PP.H1.abf 0.31%

PP.H2.abf 0.0017%

PP.H3.abf 66.9%

PP.H4.abf 32.8%

**Chromosome 11 locus (200 kb target window).**

**Comparison: Our data (25(OH)D) vs *CYP2R1* gene expression**

Probability for each scenario (%)

nsnps 52 (n)

PP.H0.abf 1.9e-06%

PP.H1.abf 0.13%

PP.H2.abf 3.9e-05%

PP.H3.abf 2.6%

PP.H4.abf 97.3%

**Comparison: Our data (25(OH)D) vs *PDE3B* gene expression**

Probability for each scenario (%)

nsnps 48 (n)

PP.H0.abf 9.4e-07%

PP.H1.abf 0.066%

PP.H2.abf 1.2e-04%

PP.H3.abf 8.3%

PP.H4.abf 91.7%

**Comparison: Our data (25(OH)D) vs *RRAS2* gene expression**

Probability for each scenario (%)

nsnps 41 (n)

PP.H0.abf 2.5e-05%

PP.H1.abf 1.7%

PP.H2.abf 4.2e-05%

PP.H3.abf 2.8%

PP.H4.abf 95.4%

A caveat with this method is that we need to make the assumption that one single variant is causing the association signals seen for each trait. We also, in principle, assume that this causative variant is included in the dataset/analysis. The precision of the method is also dependent on the number/density of SNPs used as input. Because our study is low-powered we have only used SNPs significantly associated to the trait (FDR ≤0.05) as input, to make the dataset less noisy. However, precision is also lost due to the low number of SNPs to analyze.

**References**

1. Giambartolomei C, Vukcevic D, Schadt EE, Franke L, Hingorani AD, Wallace C, et al. Bayesian Test for Colocalisation between Pairs of Genetic Association Studies Using Summary Statistics. PLoS genetics. 2014;10(5).
