# Supplementary material for: Genetic variation in GC and CYP2R1 affects 25-hydroxyvitamin D concentration and skeletal parameters: A genome-wide association study in 24-month-old Finnish children
Source: PLoS Genet. 2019 Dec 16;15(12):e1008530. doi: 10.1371/journal.pgen.1008530 (PMC6936875; doi:10.1371/journal.pgen.1008530)
Supplement: S2 Appendix — (DOCX) [file pgen.1008530.s008.docx]

S2 Appendix

**Genetic variation in *GC* and *CYP2R1* affects 25(OH)D concentration and skeletal parameters: A genome-wide association study in 24-month-old Finnish children**

Kämpe AJ MD ^1,2^, Enlund-Cerullo M MD ^3,4,5^, Valkama S MD ^3,5^, Holmlund-Suila E ^3^, MD PhD, Rosendahl J ^3,5^ MD PhD, Hauta-alus H MSc ^3,5^, Pekkinen M PhD ^3,4,5^, Andersson S MD PhD ^3^, Mäkitie O MD PhD^1,2,3,4,5^

Affiliations

^1^ Department of Molecular Medicine and Surgery, Karolinska Institutet, Stockholm, Sweden.

^2^ Department of Clinical Genetics, Karolinska University Hospital, Stockholm, Sweden

^3^ Children’s Hospital, Pediatric Research Center, University of Helsinki and Helsinki University Hospital, Helsinki, Finland

^4^ Folkhälsan Research Center, Helsinki, Finland

^5^ Research Program for Clinical and Molecular Metabolism, Faculty of Medicine, University of Helsinki, Finland

**Genome-wide association test – Complete workflow**

**Quality control**

Software used: Plink 1.9 ^(1, 2)^, KING^(3)^

*Remove samples with bad genotyping quality:*

plink --bfile VIDI_928_samples \

--mind 0.05 \

--make-bed \

--out VIDI_921_samples

= A total of 7 samples were excluded due to bad data quality (0.75%)

---------------------------------------

*Sex check:*

plink --bfile VIDI_921_samples --indep-pairphase 20000 2000 0.5 --chr 23-24

wait

plink --bfile VIDI_921_samples --extract plink.prune.in --make-bed --out ld_pruned_xy

wait

plink --bfile ld_pruned_xy --impute-sex 0.8 0.9 --make-bed --out sex_check_all_samples

---------------------------------------

R plot of F-statistic.

*There is a clear separation of female and male samples using the F-statistic (X chromosome homozygosity estimate). Only one sample (double red circles) have been inferred to the opposite sex.*

Workflow continues

---------------------------------------

*Remove the sample that failed sex check:*

plink --bfile VIDI_921_samples \

--remove samples_that_failed_sex_check.txt \

--make-bed \

--out VIDI_920_samples

---------------------------------------

The software KING was used to estimate kinship and check for duplicate samples. The algorithm is the same that is incorporated in vcftools –relatedness2 ^(4)^ . However, none of our samples had in-between kinship coefficients large enough to indicate a relationship of 1 degree relative or a pair of monozygotic twins/duplicate sample. None of the samples were excluded in this step.

---------------------------------------

**Ancestry inference:**

Software: TRACE, BEDTools, GenotypeHarmonizer

Ancestry inference was performed using the program TRACE that is contained within the LASER suite ^(5, 6)^. A 4-dimension reference space was created using the European subset of the 1000 genomes data phase 3, in total 503 individuals, of which 99 individuals are of Finnish descent. For every individual in our dataset, a 20-dimension map was created by applying a principal component analysis on each individual together with the reference population. The individuals in our dataset were then projected from the 20-dimensional map in to the 4-dimensional reference space. The LASER suite comes with a precomputed reference space for a worldwide population of 938 individuals from Human Genome Diversity Project^(7)^, but without Finnish individuals. However, the 632 958 SNPs used for the precomputed reference data had been carefully selected to be informative, strand checked and bi-allelic. We therefore intersected the 1000 genomes data with the precomputed HGDP reference SNPs using BEDTools ^(8)^. In total 114 348 SNPs overlapped all 3 datasets (HGDP reference, 1000 genomes data, our dataset), these SNPs were then later used for the ancestry inference. Before the analysis was performed, our genotype data was harmonized with the 1000 genomes dataset using Genotype Harmonizer ^(9)^, to resolve strand issues and see to that reference and alternative alleles are set to the same across both datasets. All samples in our dataset that were further away from the mean value than 4 SD (for PCs 1-4) from the 99 Finnish reference individuals were excluded. This arbitrary cutoff was set graphically, but are more conservative then recommended by the authors ^(5)^. In total 22 samples were excluded because they were deemed, genetically, to be too far from the Finnish reference individuals (S3 Fig).

**Imputation**

Software: Plink 1.9, SHAPEIT2, IMPUTE2, GenotypeHarmonizer.

Imputation, using the 1000 genomes phase 3 data as reference, was performed on all 898 that passed all filtering criteria. No filtering of the data was performed prior to imputation strategy that is supported by the findings of Roshyara and colleagues ^(10)^.

4) Split chromosomes

---------------------------------------

for chr in $(seq 1 22); do

var=$chr

plink --bfile GWAS_898_samples \

--chr ${var} \

--recode \

--make-bed \

--out ../split_by_chromosome/ GWAS_898_samples.chr${var} ;

done

---------------------------------------

*Estimating haplotypes (“phasing”) with SHAPEIT2*

Haplotype estimation was performed on all chromosomes with SHAPEIT2, as recommended in the IMPUTE2 documentation. No reference was used for this step because our sample size exceeding 200 individuals.

---------------------------------------

shapeit -B GWAS_898_samples.chr${var} \

-M genetic_map_chr${var}_combined_b37.txt \

-O phased_without_ref_chr${var} \

-T 8

---------------------------------------

The program Genotype harmonizer ^(9)^ was used for strand alignment and reference allele harmonization.

java -Xmx32g -jar GenotypeHarmonizer.jar \

--inputType SHAPEIT2 \

--input phased_without_ref_chr${var} \

--update-id \

--update-reference-allele \

--mafAlign 0.1 \

--min-ld 0.2 \

--keep \

--outputType SHAPEIT2 \

--output Phased_Harmonized_chr${var} \

--refType VCF \

--ref ALL.chr${var} .phase3_shapeit2_mvncall_integrated_v5.20130502.genotypes.vcf.gz

Chromosome X was phased and imputed separately and merged with the rest of the data afterwards.

**Imputation with IMPUTE2**

IMPUTE2 was used to performed the imputation step ^(11, 12)^. First the genome was split in 549 chunks approximately 5 MB in size.

impute2 -use_prephased_g \

-known_haps_g Phased_Harmonized_chr${var}.haps \

-sample_g Phased_Harmonized_chr${var}.sample \

-h 1000GP_Phase3_chr${var}.hap.gz \

-l 1000GP_Phase3_chr${var}.legend.gz \

-m genetic_map_ch1${var}_combined_b37.txt \

-int start end -Ne 20000 -allow_large_regions -o_gz -o chr${var}_chunk${number}

-seed 123

**Post imputation filtering:**

Using plink and unix commands, duplicates were removed, and only bi allelic SNPs with an only imputation calls with a very high confidence was kept (info score >0.8). This conservative filtering was chosen because of the small sample size in our study and we only wanted to keep confident genotype calls. All 549 chunks were then merged. Before association tests was performed, all SNPs with a missing genotype in more than >5% of samples, deviation for Hardy Weinberg equilibrium p < 0.00001 and a minor allele frequency <0.05 was excluded. The Hardy Weinberg equilibrium cutoff was chosen because it produced sensible qqplots when looking at the distribution of the HWE p-value [Supplemental appendix SM2]. Out of the 898 individuals eligible for the analysis, 761 of the children had 25(OH)D measurements at 24 months. After post imputation filtering the total amount of SNPs used in the analysis was 5,072,729.

**Principal component analysis**

Principal component analysis was performed on the non-imputed data using FlashPCA^(13)^

plink --bfile non_imputed_QC --indep-pairwise 1000 50 0.05 --exclude range exclude_regions.txt --out for_pruning

plink --bfile non_imputed_QC --extract for_pruning.prune.in --make-bed --out pruned

flashpca --bfile pruned --suffix flash_PCA

**Association test**

Software: Plink (v1.9)^(1)^, Trait: 25OH(D) level at 24 months. Covariables: Gender, randomization group (either 10 µg (standard) or 30 µg (treatment) of Vitamin D daily, season of vitamin D measurement and principal components 1-4.

plink --bfile ${imputed} --pheno vitD_24_months.txt --linear sex hide-covar --covar ${covars} --covar-number 1,2,3,4,5,6 --hardy --freq --ci 0.95 --out Vit_D_assoc

Genomic inflation factor (lambda): 0.9972035

QQ-plot of 25(OH)D association test (S1 Fig)

Manhattan plot of 25(OH)D association test (Fig 1)

**References**

1. Purcell S, Neale B, Todd-Brown K, Thomas L, Ferreira MAR, Bender D, et al. PLINK: A tool set for whole-genome association and population-based linkage analyses. Am J Hum Genet. 2007;81(3):559-75.

2. Package: PLINK 1.9., Author: Shaun Purcell, URL:<https://www.cog-genomics.org/plink2>

.

3. Manichaikul A, Mychaleckyj JC, Rich SS, Daly K, Sale M, Chen WM. Robust relationship inference in genome-wide association studies. Bioinformatics. 2010;26(22):2867-73.

4. Danecek P, Auton A, Abecasis G, Albers CA, Banks E, DePristo MA, et al. The variant call format and VCFtools. Bioinformatics. 2011;27(15):2156-8.

5. Wang C, Zhan X, Liang L, Abecasis GR, Lin X. Improved ancestry estimation for both genotyping and sequencing data using projection procrustes analysis and genotype imputation. Am J Hum Genet. 2015;96(6):926-37.

6. Wang C, Zhan X, Bragg-Gresham J, Kang HM, Stambolian D, Chew EY, et al. Ancestry estimation and control of population stratification for sequence-based association studies. Nat Genet. 2014;46(4):409-15.

7. Li JZ, Absher DM, Tang H, Southwick AM, Casto AM, Ramachandran S, et al. Worldwide human relationships inferred from genome-wide patterns of variation. Science. 2008;319(5866):1100-4.

8. Quinlan AR, Hall IM. BEDTools: a flexible suite of utilities for comparing genomic features. Bioinformatics. 2010;26(6):841-2.

9. Deelen P, Bonder MJ, van der Velde KJ, Westra HJ, Winder E, Hendriksen D, et al. Genotype harmonizer: automatic strand alignment and format conversion for genotype data integration. BMC Res Notes. 2014;7:901.

10. Roshyara NR, Kirsten H, Horn K, Ahnert P, Scholz M. Impact of pre-imputation SNP-filtering on genotype imputation results. BMC Genet. 2014;15.

11. Howie BN, Donnelly P, Marchini J. A flexible and accurate genotype imputation method for the next generation of genome-wide association studies. PLoS genetics. 2009;5(6):e1000529.

12. Howie B, Marchini J, Stephens M. Genotype imputation with thousands of genomes. G3 (Bethesda). 2011;1(6):457-70.

13. Abraham G, Inouye M. Fast Principal Component Analysis of Large-Scale Genome-Wide Data. PLoS One. 2014;9(4).
